# Tris(bipyridine)Metal(II)-Templated Assemblies of 3D Alkali-Ruthenium Oxalate Coordination Frameworks: Crystal Structures, Characterization and Photocatalytic Activity in Water Reduction

**DOI:** 10.3390/polym8020048

**Published:** 2016-02-15

**Authors:** Alla Dikhtiarenko, Pedro Villanueva-Delgado, Rafael Valiente, José R. García, José Gimeno

**Affiliations:** 1 Organic and Inorganic Chemistry Department, University of Oviedo-CINN, 33006 Oviedo, Spain; jgh@uniovi.es; 2 Department of Chemistry and Biochemistry, University of Bern, 3012 Bern, Switzerland; pedro.villanueva@dcb.unibe.ch; 3 MALTA Consolider Team, Department of Applied Physic, University of Cantabria, 39005 Santander, Spain; rafael.valiente@unican.es

**Keywords:** water splitting, hydrogen evolution, coordination polymers, photocatalysts

## Abstract

A series of 3D oxalate-bridged ruthenium-based coordination polymers with the formula of {[Z^II^(bpy)_3_][M^I^Ru(C_2_O_4_)_3_]}_n_ (Z^II^ = Zn^2+^ (**1**), Cu^2+^ (**3**, **4**), Ru^2+^ (**5**, **6**), Os^2+^ (**7**, **8**); M^I^ = Li^+^, Na^+^; bpy = 2,2’-bipyridine) and {[Zn^II^(bpy)_3_](H_2_O)[LiRu(C_2_O_4_)_3_]}_n_ (**2**) has been synthesized at room temperature through a self-assembly reaction in aqueous media and characterized by single-crystal and powder X-ray diffraction, elemental analysis, infrared and diffuse reflectance UV–Vis spectroscopy and thermogravimetric analysis. The crystal structures of all compounds comprise chiral 3D honeycomb-like polymeric nets of the srs-type, which possess triangular anionic cages where [Z^II^(bpy)_3_]^2+^ cationic templates are selectively embedded. Structural analysis reveals that the electronic configuration of the cationic guests is affected by electrostatic interaction with the anionic framework. Moreover, the MLCT bands gaps values for **1**–**8** can be tuned in a rational way by judicious choice of [Z^II^(bpy)_3_]^2+^ guests. The 3D host-guest polymeric architectures can be used as self-supported heterogeneous photocatalysts for the reductive splitting of water, exhibiting photocatalytic activity for the evolution of H_2_ under UV light irradiation.

## 1. Introduction

In recent years, the depletion of fossil fuels and the environmental problems caused by their combustion have stimulated research on the development of new renewable energy production technologies. So far, several approaches have been proposed in order to address this challenge. Among those explored, the system combining photocatalysts and solar energy as a clean and abundant energy resource is recognized to be of great promise. Currently, enormous attention has been paid to photocatalytic hydrogen production from water, which is a promising way to produce hydrogen as a potential clean energy source [1,2]. In this line, the hybridization of organic and inorganic materials opens up a new field in the design and preparation of applicable photocatalysts for water splitting reaction by the integration of useful organic and inorganic characteristics within a single composite [3,4].

In this sense, metal-organic frameworks (MOFs) and coordination polymers (CPs) [5], which are organic–inorganic hybrid materials consisting of organic linkers and metal centers, clusters or metal-oxo clusters, have received great interest due to properties, such as extremely high surface areas, well-ordered porous architectures and structural designability [6,7]. Taking advantage of these interesting properties, MOFs/CPs are widely studied for many potential applications, from gas storage to molecular sieving, ion conductivity and catalysis [8,9,10,11,12,13]. Additionally, in recent years, an increasing number of studies has demonstrated that MOFs serve as a platform for integrating different functional components to achieve light harvesting [14,15,16] and to drive various photocatalytic reactions [17,18], such as carbon dioxide reduction to CO [19,20], formic acid [21,22,23] or methanol [24,25], synthesis of metallic nanoparticles [26] or metallic nanostructures for lithographic pattering [27], oxidation of organic compounds [28,29,30,31,32], degradation of organic dyes [33,34,35,36,37] and various organic transformations [19,38,39,40]. Compared to the other photocatalytic systems, the MOFs photocatalysts have advantages in that the variety of combinations of bridging organic linkers [41,42] and metallic centers [43,44] allows for the fine-tuning and rational design of these photocatalysts at the molecular level. In this context, recent synthetic achievements have been delivered to robust MOFs/CPs displaying photocatalytic activity in photocatalytic water splitting reaction towards H_2_ production [45,46].

In 2009, Kataoka *et al.* firstly applied ruthenium-based MOFs as a heterogeneous catalyst for photo-promoted H_2_ production [47]. Under VIS light irradiation, the photocatalytic system containing [Ru^II,III^_2_(BDC)_2_BF_4_]_n_ MOF (BDC = benzene-1,4-dicarboxylate) acting as catalysts, [Ru(bpy)_3_]^2+^ (bpy = 2,2’-bipyridine) as photosensitizer, EDTA (ethylenediaminetetraacetate) and MV^2+^ (*N*,*N*’-dimethyl-4,4’-bipyridinium) as electron donors was able to photo-split water molecules, generating H_2_ with high rates. Later, this study was extended on a series of analogous [Ru^II,III^_2_(BDC)_2_X]_n_ (X = BF^4−^, Cl^−^, Br^−^) MOFs based on the diruthenium paddle-well structural units, with an attempt to determine the effect of the incorporated anion on the photocatalytic behavior of the materials [48]. Among more recent studies, the photocatalytic activity of NH_2_–MOF–Ti [49,50] and NH_2_–UiO-66(Zr) [51] was improved through post-synthetic deposition within the framework of Pt nanoparticles, which in turn behave as co-catalysts in a reductive water splitting reaction. Similarly, the MIL-101(Cr) MOF with embedded CdS nanoparticles shows high catalytic activity towards H_2_ generation upon VIS light [52]. Moreover, several photocatalytic MOFs for hydrogen evolution were prepared via post-modification of organic linkers incorporating photosensitizer molecule or a photoactive complex, such as in the case of the UiO-66(Zr) framework sensitized with rhodamine B [53], UiO-67(Zr) with the target [Ir(ppy)_2_(bpy)]^+^ (ppy = 2-phenylpyridine, bpy = 2,2’-bipyridine) complex [54], MOF-253(Al) with the post-synthetically-immobilized Pt-complex [55] or UiO-66(Zr) with [[FeFe]-(dcbdt)(CO)_6_] (dcbdt = 1,4-dicarboxylbenzene-2,3-dithiolate) -loaded functional groups [56]. Moreover, due to the complex, laborious and multistep way of post-synthetic functionalization, several photocatalytically-active MOFs/CPs for hydrogen evolution were obtained through the easiest one-pot syntheses; for instance, {[Ln_2_Cu_5_(OH)_2_(pydc)_6_(H_2_O)_8_]·I_8_}_n_ (Ln = Sm, Eu, Gd and Tb) MOF templated by iodine anions [57], polyoxometalate-based {(TBA)_2_[Cu^II^(BBTZ)_2_(*x*-Mo_8_O_26_)]}_n_ (TBA = tetrabutylammonium cation; BBTZ = 1,4-bis(1,2,4-triazol-1ylmethyl)-benzene; *x* = β and α) anionic frameworks [58] or porphyrin-based {[Al(OH)]_2_H_2_TCPP(DMF_3_-(H_2_O)_2_}_n_ (H_2_TCPP = tetra(4-carboxyl-phenyl)porphyrin) [59]. Recently, Nasalevich *et al.* reported another approach for efficient visible light H_2_ evolution via a ship in a bottle strategy [60].

Regarding the benefits of one-pot synthesis paths for the preparation of photocatalytically-active MOF/CPs and taking into account the potential disadvantages of post-synthetic approaches, such as inhomogeneous distribution and functionalization degrees, we envisioned that known host-guest oxalate-bridged 3D frameworks with the general formula of {[Z^II^(bpy)_3_][M^I^M^III^(C_2_O_4_)_3_]}_n_ (where the Z^II^:M^I^:M^III^ metal combinations known are Fe^2+^:Li^+^:Cr^3+^, Fe^2+^:Na^+^:Cr^3+^, Fe^2+^:Li^+^:Fe^3+^, Fe^2+^:Na^+^:Fe^3+^, Zn^2+^:Na^+^:Al^3+^, Zn^2+^:Na^+^:Cr^3+^, Ru^2+^:Na^+^:Al^3+^, Ru^2+^:Li^+^:Cr^3+^, Ru^2+^:Na^+^:Cr^3+^, Ru^2+^:Na^+^:Rh^3+^, Co^2+^:Na^+^:Cr^3+^, Co^2+^:Li^+^:Cr^3+^, Ni^2+^:Na^+^:Al^3+^, Os^2+^:Na^+^:Al^3+^) [61,62,63,64,65,66,67,68,69,70], {[Z^II^(bpy)_3_](H_2_O)[M^I^M^III^(C_2_O_4_)_3_]}_n_ (where the Z^II^:M^I^:M^III^ metal combinations known are Ni^2+^:Li^+^:Cr^3+^ and Ru^2+^:Li^+^:Cr^3+^) [71], {[Z^III^(bpy)_3_](X)[M^I^M^III^(C_2_O_4_)_3_]}_n_ (where X = ClO_4_^−^, PF_6_^−^, BF_4_^−^; bpy = 2,2’-bipyridine; the Z^III^:M^I^:M^III^ metal combinations known are Rh^3+^:Na^+^:Cr^3+^, Rh^3+^:Na^+^:Al^3+^, Rh^3+^:Na^+^:Rh^3+^, Cr^3+^:Na^+^:Cr^3+^, Cr^3+^:Na^+^:Al^3+^, Cr^3+^:Na^+^:Rh^3+^, Co^3+^:Na^+^:Cr^3+^) [62,63,70,72,73,74,75,76] could be positioned as deserving competitors along with those functional MOFs that encapsulate photoactive guest molecules in the pores of the framework [77]. In this class of compounds, the [Z^II^(bpy)_3_]^2+^ cations tightly fit into vacant cavities provided by the three-dimensional anionic {[M^I^M^III^(C_2_O_4_)_3_]^2−^}_n_ network. Thereby, tris-bipyridine complexes are quantitatively and homogeneously distributed within the polymeric framework. Moreover, the chemical variation and combination of the metal ions in the oxalate backbone, as well as in the tris-bipyridine cation offer unique opportunities for the rational design of a photoactive coordination polymer with the desired photochemical and photophysical properties, such as light-induced electron transfer and excitation energy transfer in the solid state.

Thus, herein, we present the synthesis of a series of new three-dimensional ruthenium-based oxalate-bridged anionic networks {[M^I^Ru^III^(C_2_O_4_)_3_]^2−^}_n_ (M^I^ = Na^+^, Li^+^) in which the large honeycombed channels are occupied by [Z^II^(bpy)_3_]^2+^ (bpy = 2,2´-bipyridine, Z^II^ = Zn^2+^, Cu^2+^, Ru^2+^, Os^2+^) cationic templates. In addition to a thorough structural characterization, we demonstrate the high photocatalytic activity of these structured solids.

## 2. Materials and Methods

### 2.1. Materials

The complexes [Z^II^(bpy)_3_](ClO_4_)_2_ (where Z^II^ = Zn^2+^, Cu^2+^, Ru^2+^), [Os^II^(bpy)_3_](PF_6_)_2_ and K_3_[Ru(C_2_O_4_)_3_]·4.5H_2_O were prepared according to the literature methods [78,79,80]. The other chemicals are commercially available and were used as purchased.

### 2.2. Synthesis of the {[Z^II^(bpy)_3_][NaRu(C_2_O_4_)_3_]}_n_ (Z^II^ = Zn^2+^ (**1**), Cu^2+^ (**3**), Ru^2+^ (**5**), Os^2+^ (**7**)), {[Z^II^(bpy)_3_][LiRu(C_2_O_4_)_3_]}_n_ (Z^II^ = Cu^2+^ (**4**), Ru^2+^ (**6**), Os^2+^ (**8**)) and {[Zn(bpy)_3_](H_2_O)[LiRu(C_2_O_4_)_3_]}_n_ (**2**) Series of Compounds

The synthesis process was performed in accordance with a previously-published procedure for the {[Fe^II^(bpy)_3_][M^I^Cr(C_2_O_4_)_3_]} (where M^I^ = Na^+^, Li^+^) compounds [61] introducing the [Ru(C_2_O_4_)_3_]^3−^ moiety instead of [Cr(C_2_O_4_)_3_]^3−^. In a typical synthesis, 141 mg (0.25 mmol) of K_3_[Ru(C_2_O_4_)_3_]·4.5H_2_O and 30 mg (0.5 mmol) of NaCl or 20 mg (0.5 mmol) of LiCl were dissolved in 5 mL of water, and 0.25 mmol of the [Z^II^(bpy)_3_](ClO_4_)_2_ salt ([Zn^II^(bpy)_3_](ClO_4_)_2_, 183 mg; [Cu^II^(bpy)_3_](ClO_4_)_2_, 183 mg; [Ru^II^(bpy)_3_](ClO_4_)_2_, 192 mg; [Os^II^(bpy)_3_](PF_6_)_2_, 237 mg) dissolved in a water/ethanol mixture were added dropwise; after few minutes, precipitates appeared, and the suspensions were stirred for 1 h. The resulting precipitates were filtered, washed with ethanol and air dried.

Yellow precipitates of **1** and **2** yield 90% and 74%, respectively. Anal. Calc. for C_36_H_24_N_6_NaO_12_RuZn (**1**): C, 46.85%; H, 2.60%; N, 9.11%. Found: C, 47.0%; H, 2.85%; N, 9.1%. Anal. Calc. for C_36_H_26_LiN_6_O_13_RuZn (**2**): C, 46.75%; H, 2.81%; N, 9.09%. Found: C, 46.8%; H, 2.9%; N, 9.1%.

Greenish precipitates of **3** and **4** yield 81% and 77%, respectively. Anal. Calc. for C_36_H_24_CuN_6_NaO_12_Ru (**3**): C, 46.94%; H, 2.83%; N, 9.13%. Found: C, 50.0%; H, 2.9%; N, 9.2%. Anal. Calc. for C_36_H_24_CuLiN_6_O_12_Ru (**4**): C, 47.78%; H, 2.87%; N, 9.29%. Found: C, 47.8%; H, 2.9%; N, 9.3%.

Red-orange precipitates of **5** and **6** yield 62% and 78%, respectively. Anal. Calc. for C_36_H_24_N_6_NaO_12_Ru_2_ (**5**): C, 45.11%; H, 2.51%; N, 8.77%. Found: C, 45.0%; H, 2.7%; N, 8.8%. Anal. Calc. for C_36_H_24_LiN_6_O_12_Ru_2_ (**6**): C, 45.87%; H, 2.55%; N, 8.92%. Found: C, 46.5%; H, 2.8%; N, 9.2%.

Dark green precipitates of **7** and **8** yield 54% and 68%, respectively. Anal. Calc. for C_36_H_24_N_6_NaO_12_OsRu (**7**): C, 41.26%; H, 2.29%; N, 8.02%. Found: C, 41.4%; H, 2.3%; N, 8.2%. Anal. Calc. for C_36_H_24_LiN_6_O_12_OsRu (**8**): C, 41.91%; H, 2.33%; N, 8.15%. Found: C, 42.1%; H, 2.3%; N, 8.3%.

### 2.3. X-Ray Structure Determinations

Tetrahedral-shaped single crystals of Compounds **1**−**8** (Figure S1) were selected for single-crystal X-ray diffraction analyses. The intensity data were collected at room temperature on an Oxford-Gemini X-ray diffractometer using for Compounds **2** and **4** graphite-monochromatic Mo-Kα (λ = 0.71073 Å) and for **1**, **3**, **5**−**8**, Cu-Kα (λ = 1.54184 Å) radiation. The CrysAlisPro software was used for cell refinement and data reduction. Images were collected at a 55-mm fixed crystal-detector distance, using the oscillation method, with 1 oscillation and variable exposure time per image. The structures were solved by direct methods using the SIR92 program [81]. The refinement was performed by SHELX-97 using full-matrix least squares on *F*^2^ [82]. All non-H atoms were anisotropically refined. The hydrogen atoms of the 2,2’-bipyridine ligand were placed geometrically, and the hydrogen atoms of the water molecule in Compound **2** could not be located, but were included in the formula. Flack’s absolute parameter (*x*) was used to determine the space group of compounds [83]. Crystallographic data for **1**−**8** (CCDC#1404961–1404964, #1404970–1404973) have been deposited with Cambridge Crystallographic Data Centre. The detailed crystallographic data are summarized in Table S1 (Supplementary Materials). Topological and geometrical analysis of **1**−**8** was obtained using TOPOS 4.0 software [84]. X-ray powder diffraction patterns were collected with a X’Pert Philips X-ray diffractometer (CuKα radiation, λ = 1.5418 Å) at room temperature. The powder diffraction patterns indicate that all compounds are isostructural and show analogous patterns to the simulated patterns from the atomic coordinates of the crystal structures of **1**−**8** (Figures S2–S5, Supplementary Materials).

### 2.4. Characterization Methods

The IR spectra were recorded on a Bruker Tensor-27 spectrophotometer as KBr pellets in the 4000–500 cm^−1^ region. Microanalyses (C, H, N) were carried out by the use of a Perkin-Elmer model 2400B elemental analyzer. X-ray microanalysis (SEM/EDX) confirmed the ratio Ru:Z^II^ to be 1:1 (Z^II^ = Zn^2+^, Cu^2+^, Os^2+^), by using JEOL JSM-6100 scanning microscopy (SEM) coupled with an INCA Energy-200 dispersive X-ray microanalysis system (EDX) with a PentaFET ultrathin window detector. As shown in Figure S6 (Supplementary Materials), the microcrystalline texture of the samples consists of microcrystals that repeat the same habit as those obtained single crystals, indicating that powder products have been obtained as pure phases. A Mettler-Toledo TGA/SDTA851 was used for the thermal analyses in a nitrogen and air dynamic atmosphere (50 mL/min) at a heating rate of 10 °C/min. Approximately 10 mg of powder sample were thermally treated, and blank runs were performed. A Pfeiffer Vacuum TermoStar™ GSD301T mass spectrometer was used to determine the evacuated vapors. The masses 15 (NH_3_), 18 (H_2_O), 44 (CO_2_) and 46 (NO_2_) were tested by using a detector C-SEM, operating at 1200 V, with a time constant of 1 s. A Cary 6000i (Varian) spectrophotometer was used to measure diffuse reflectance spectra in the range 200–1800 nm using a polytetrafluoroethylene (PTFE)-coated integrating sphere.

### 2.5. Photocatalytic Hydrogen Evolution

Reactions were carried out at room temperature in a 100-mL gastight cell that was custom-designed in order to allow purging and irradiation of the suspension. The gastight cell was a 100-mL two-necked, flat-bottomed flask with a water refrigerator. The cell volume was 100 mL, of which gases occupied 83 mL. In each experiment, 10 μmol of heterogeneous catalyst were dispersed in a mixture containing 10 mL H_2_O and 7 mL TEA (triethylamine). Reaction mixtures were deoxygenated with three cycles of evacuation and purging with argon. The samples’ solutions were illuminated with UV light at room temperature by a 500-W mercury lamp (HELIOS ITALQUARTZ Apparatus, Model UV50F–85P503I5, ≤366 nm) for 12 h. During reaction, magnetic stirring was used to prevent sedimentation of the catalyst. For experiments performed with visible light irradiation, the xenon lamp (150 W, ≥417 nm) was used as the light source. Reaction products were analyzed by mass spectrometry taking regular aliquots (0.5 mL) of the reactor headspace gas through a septum using a gastight syringe. Mass spectrometry analyses were performed using an OmniStar™ (Pfeiffer Vacuum) gas analysis module connected to AutoChem II 2920 (Micromeritics) catalyst characterization system. A cold trap was used with Ar as the carrier gas. Each gas aliquot was quantified using the calibration graph, which had been previously obtained using standard 10% (*v*/*v*) H_2_ in Ar and 5% (*v*/*v*) O_2_ in He gas mixtures (Air Liquid, Paris, France).

## 3. Results and Discussion

### 3.1. Crystal Structures

Compounds **1**‒**8** present the 3D three-connected decagon oxalate-bridged anionic network {[M^I^Ru(C_2_O_4_)_3_]^2−^}_n_ (M^I^ = Na^+^, Li^+^), with the cationic complex [Z^II^(bpy)_3_]^2+^ (where Z^II^ = Zn^2+^, Cu^2+^, Ru^2+^, Os^2+^; bpy = 2,2´-bipyridine) acting as the template. The single-crystal X-ray analysis of ruthenium-based 3D oxalate bridged polymers reveals that the CPs **1**, **3**−**8** are isostructural with the {[Z^II^(bpy)_3_][M^I^M^III^(C_2_O_4_)_3_]}_n_ (Z^II^ = Co^2+^, Zn^2+^, Ni^2+^, Fe^2+^, Ru^2+^; M^I^ = Na^+^, Li^+^; M^III^ = Rh^3+^, Ru^3+^, Al^3+^, Cr^3+^, Fe^3+^) family of compounds [61,62,63,64,65,66,67,68,69,70]. However, Compound **2** is isomorphic with the {[Z^II^(bpy)_3_](H_2_O)[LiCr(C_2_O_4_)_3_]}_n_ (Z^II^ = Ni^2+^, Ru^2+^) family of 3D oxalate networks [71].

**Figure 1:**
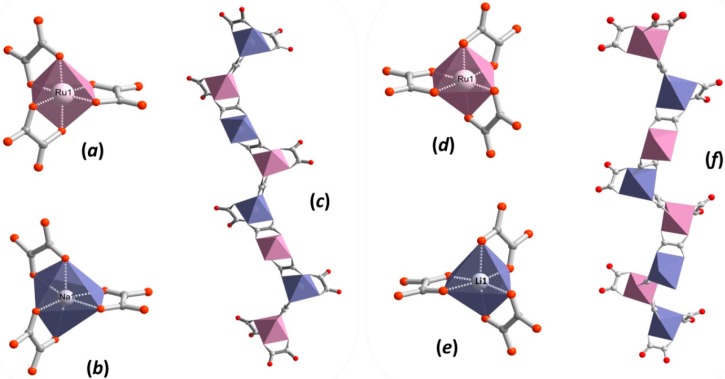
Representation of octahedral coordination environments of (**a**) Ru^III^ and (**b**) Na^I^ in **1** exhibiting Λ-conformation with the corresponding (**c**) left-handed helix substructure formed. Representation of octahedral coordination environments of (**d**) Ru^III^ and (**e**) Li^I^ in **1** exhibiting Δ-conformation with the corresponding (**f**) right-handed helix substructure formed. Red and grey spheres represent oxygen and carbon atoms, respectively.

The detailed crystal data and structure determination parameters of ruthenium-based coordination polymers **1**–**8** are summarized in Table S1. The CPs **1**–**8** crystalize in the cubic chiral space group *P*2_1_3 with the asymmetric unit consisting of a complete oxalate ligand, the Ru^3+^ and Na^+^/Li^+^ ions of the anionic network, the Z^II^ metal center (Z^II^ = Zn^2+^, Cu^2+^, Ru^2+^, Os^2+^) and the complete bpy ligand of the cationic template (Figure S7a). Each Ru^3+^ and Na^+^/Li^+^ ion is surrounded by six oxygen atoms of the oxalate ligand forming a distorted octahedral coordination environment (Figure 1a,b,d,e) with the mean Ru−O and Na/Li−O bond lengths, which are within the range observed for analogous compounds [65].

Selected bond distances and distortion parameters of Ru^III^, Z^II^ and M^I^ coordination environments for Compounds **1**−**8** are given in Table 1. Interestingly, the {Ru(C_2_O_4_)_3_} and {M^I^(C_2_O_4_)_3_} structural units (SBU) manifest the same Δ or Λ-configuration in the chiral 3D anionic networks (Figure 1a,b,d,e). Thus, Compounds **1**, **4**‒**6** and **8** build SBU with the Λ-form configuration, while **2**–**3** and **7** are constructed with the Δ-form.

**Figure 2:**
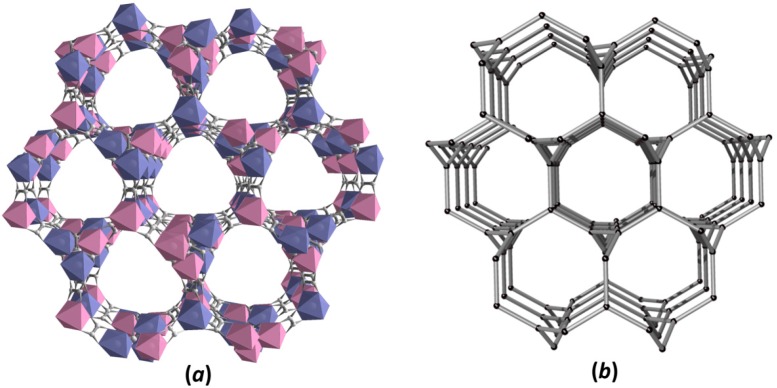
The 3D three-connected decagon anionic network {[M^I^Ru(C_2_O_4_)_3_]^2−^}_n_ (M^I^ = Na, Li): (**a**) view of honeycombed channels along the [111] direction and (**b**) its simplified topological representation, where black spheres represent a node of equivalent Ru^III^ and M^I^ centers.

In this type of structure, the oxalate ligand exhibiting µ-coordination mode (Figure S7b) links in an alternate manner Ru^3+^ and Na^+^/Li^+^ metal centers to form helical substructures, where Ru···Na/Li distances ranged from 5.46–5.63 Å. As shown in Figure 1c,f, helical substructures with three-fold axis interpretation spread along the *b*-axis and, depending on the {Ru(C_2_O_4_)_3_} and {M^I^(C_2_O_4_)_3_} SBUs’ conformations (Δ or Λ), exhibit left- or right-handed rotation. Repeatedly connected adjacent helices form a porous anionic 3D framework with honeycomb-like channels running along the [111] crystallographic direction (Figure 2a). According to topological analysis performed using TOPOS 4.0 software [84], resulting 3D anionic networks are three-connected uninodal nets with a 10^3^-*a* array topology (Figure 2b; also denoted as the srs-type net) [85].

**Table 1:** Selected bond length (Å) for {[Z^II^(bpy)_3_][NaRu(C_2_O_4_)_3_]}_n_ (Z^II^ = Zn^2+^ (**1**), Cu^2+^ (**3**), Ru^2+^ (**5**), Os^2+^ (**7**)), {[Zn^II^(bpy)_3_](H_2_O)[LiRu(C_2_O_4_)_3_]}_n_ (**2**) and [Z^II^(bpy)_3_][LiRu(C_2_O_4_)_3_]}_n_ (Z^II^ = Cu^2+^ (**4**), Ru^2+^ (**6**), Os^2+^ (**8**)) coordination polymers; the configuration and structural distortion parameters of [Z^II^(bpy)_3_]^2+^ (Z^II^ = Zn^2+^, Cu^2+^, Ru^2+^, Os^2+^) guests compared to the corresponding [Z^II^(bpy)_3_]^2+^ cation in salts ^1^.

Compound	1–2 [Zn(bpy)_3_]^2+^		3–4 [Cu(bpy)_3_]^2+^			5–6 [Ru(bpy)_3_]^2+^			7–8 [Os(bpy)_3_]^2+^	
Bonds	M^I^ = Na	M^I^ = Li (H_2_O)	M^I^ = Na	M^I^ = Li		M^I^ = Na	M^I^ = Li		M^I^ = Na	M^I^ = Li
Ru–O1	2.013(7)	2.047(3)	2.017(3)	2.034(2)		2.021(3)	2.029(2)		2.027(5)	2.038(4)
Ru–O2	2.036(5)	2.050(3)	2.030(3)	2.044(2)		2.023(3)	2.043(3)		2.045(5)	2.049(4)
M^I^–O3	2.336(9)	2.110(9)	2.336(4)	2.136(6)		2.319(4)	2.214(4)		2.306(6)	2.140(9)
M^I^–O4	2.375(9)	2.232(9)	2.339(4)	2.235(5)		2.330(4)	2.220(5)		2.312(6)	2.236(9)
Z^II^–N1	2.126(8)	2.028(4)	2.116(4)	2.100(3)		2.059(3)	2.052(2)		2.057(5)	2.060(4)
Z^II^–N2	2.141(8)	2.034(4)	2.125(3)	2.101(3)		2.063(3)	2.059(2)		2.064(5)	2.062(4)
Z^II^–N_av_	2.133(6)	2.031(2)	2.121(4)	2.100(4)		2.061(2)	2.055(3)		2.061(3)	2.061(1)
Δ /Λ form	Λ	Δ	Δ	Λ		Λ	Λ		Δ	Λ
^2^ σ^2^	68.0	37.3	60.9	56.2		49.1	48.5		53.9	53.5
^3^ λ	1.2 × 10^−5^	2.2 × 10^−6^	4.5 × 10^−6^	5.0 × 10^−8^		9.4· × 10^−7^	2.9 × 10^−6^		2.8 × 10^−6^	2.3 ×·10^−7^
Bond length and distortion parameters of [Z^II^(bpy)_3_]^2+^ cation in salts ^1^										
**Complex**	**[Zn(bpy)_3_]^2+^**		**[Cu(bpy)_3_]^2+^**		**[Ru(bpy)_3_]^2+^**			**[Os(bpy)_3_]^2+^**		
Z^II^–N_rang._	2.110(5)–2.240(3)		2.020(2)–2.454(2)		2.056(1)–2.060(1)			2.062(1)–2.062(1)		
Z^II^–N_av_	2.159(10)		2.136(11)		2.058(2)			2.062		
^2^ σ^2^	95.6		85.1		57.6			63.9		
^3^ λ	4.8 × 10^−4^		5.7 × 10^−3^		6.8 × 10^−7^			3.7 × 10^−6^		

^1^ The M^II^–N bonds lengths for [Zn(bpy)_3_](ClO_4_)_2_ [86], [Cu(bpy)_3_](ClO_4_)_2_ [87], [Ru(bpy)_3_](ClO_4_)_2_ [88] and [Os(bpy)_3_](PF_6_)_2_ [89] salts were taken from the published crystallographic data. ^2^ The bond angle variance: $\sigma^{2}=\frac{1}{11}\underset{n=1,12}{\sum }{\left(\theta_{n}-90{}^{\circ}\right)}^{2}$, where θ_n_ is one of the twelve N–Z^II^–N angles in the coordination sphere [90]. ^3^ The mean quadratic elongation: $\lambda =\frac{1}{6}\underset{n=1,6}{\sum }{\left[\frac{\left(d_{n}-\langle d\rangle \right)}{\langle d\rangle }\right]}^{2}$, where ‹*d*› and *d*_n_ are the mean Z^II^–N bond length and the six Z^II^–N bond lengths in coordination polyhedra, respectively [91].

**Figure 3:**
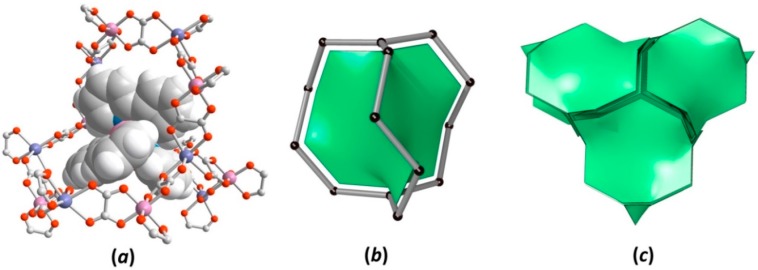
(**a**) Perspective view of the [Z^II^(bpy)_3_]^2+^ (Z^II^ = Zn^2+^, Cu^2+^, Ru^2+^, Os^2+^) complex hosted in the anionic three-fold cage. (**b**) Simplified topological representation of the anionic cage (black nodes are Ru^III^ and M^I^ metal centers; grey rods are the oxalate ligand) with the *srs* triangular tile. (**c**) Tile packing, which reconstructs the cage structure in the 3D honeycomb-like framework.

In fact, the {[M^I^Ru(C_2_O_4_)_3_]^2−^}_n_ (M^I^ = Na^+^, Li^+^) anionic frameworks are cage-like structures with three-fold cavities formed as a result of helical substructure interconnection. The tris-chelating cationic [Z^II^(bpy)_3_]^2+^ (where Z^II^ = Zn^2+^, Cu^2+^, Ru^2+^, Os^2+^; bpy = 2,2’-bipyridine) complex acting as the charge balanced template fits the large anionic cavities in a specific and highly symmetrical manner (Figure 3a). Interestingly, the cationic entity acts as a structural (appropriate size/shape), stoichiometric and chiral template, which repeats the homochiral conformational characteristics (Δ or Λ), such as SBUs in the polymeric network, resumed in Table 1. The role of bulky [Z^II^(bpy)_3_]^2+^ cations in oxalate-based anionic coordination arrays has been previously investigated and has a significant effect on the network structure formation [72,92].

For the sake of topological simplification of the 3D framework structures, the anionic cavities are generalized as the self-dual natural tile characteristic for 10,3-net topologies and can be described as a triangle vertex figure with 14 vertices and three faces (Figure 3b). As illustrated in Figure 3c, the [10^3^] tiles sharing one face reconstruct porous spaces of the anionic network to form the 3D honeycombed architecture.

**Figure 4:**
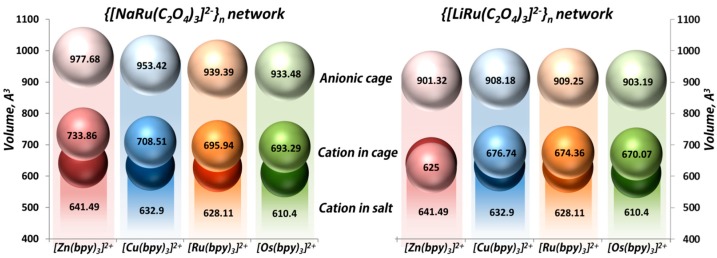
Representation of accessible volumes of anionic cages in {[NaRu(C_2_O_4_)_3_]^2−^}_n_ (**right**) and networks {[LiRu(C_2_O_4_)_3_]^2−^}_n_ (**left**), the volumes of [Z^II^(bpy)_3_]^2+^ (Z^II^ = Zn^2+^, Cu^2+^, Ru^2+^,Os^2+^) cationic complexes in their salt forms (darker spheres) and incorporated in corresponding 3D polymeric nets (medium spheres). The volume calculation for the [Z^II^(bpy)_3_]^2+^ complex in salt forms has been performed using the published crystallographic data [86,87,88,89].

Applying the models of Voronoi–Dirichlet polyhedra [93], an accessible volume of three-fold anionic cages in {[M^I^Ru(C_2_O_4_)_3_]^2−^}_n_ (M^I^ = Na^+^, Li^+^) nets, the volume of cation [Z^II^(bpy)_3_]^2+^ (Z^II^ = Zn^2+^, Cu^2+^, Ru^2+^ and Os^2+^) incorporated in the networks and their volume in free salts were calculated and summarized in Figure 4. The volume of anionic cages in the {[NaRu(C_2_O_4_)_3_]^2−^}_n_ framework are slightly bigger than those in the {[LiRu(C_2_O_4_)_3_]^2−^}_n_, which is caused by the difference between the ionic radii of Na and Li metal centers incorporated in the framework. Notably, the cationic template [Z^II^(bpy)_3_]^2+^ (where Z^II^ = Zn^2+^, Cu^2+^, Ru^2+^, Os^2+^) selectively residing in the anionic cages of **1**, **3**–**8** undergoes a 6.9%–14.4% expansion compared to the corresponding cationic complex in the free salt forms.

Compound **2** is an outstanding case of this family, where the volume of the [Zn(bpy)_3_]^2+^ template is smaller (2.6%) than in the free salt, in contrast to **1**, **3**–**8**. Such a difference is related to the incorporation of additional water molecules (one per unit formula: {[Zn^II^(bpy)_3_](H_2_O)[LiCr(C_2_O_4_)_3_]}_n_) and was observed in analogous compounds {[Z^II^(bpy)_3_](H_2_O)[LiCr(C_2_O_4_)_3_]}_n_ (Z^II^ = Ni^2+^, Ru^2+^) [71] and {[Z^III^(bpy)_3_](X)[NaM^III^(C_2_O_4_)_3_]}_n_ (M^III^ = Cr^3+^, Al^3+^, Rh^3+^; Z^III^ = Cr^3+^, Rh^3+^, Co^3+^; X = ClO_4_^−^, PF_6_^−^) [72,73,74,75,76], The special packing arrangement of [Z^III^(bpy)_3_]^3+^ or [Z^II^(bpy)_3_]^2+^ cations creates cubic-shaped cavities able to encapsulate small anions (ClO_4_^−^ or PF_6_^−^) or neutral molecules (H_2_O). In the case of **2**, three pairs of parallel aligned, adjacent bpy ligands, perpendicularly oriented to each other, form the cubic-shaped vacancies in which the water molecules reside with full occupancy of this site.

**Figure 5:**
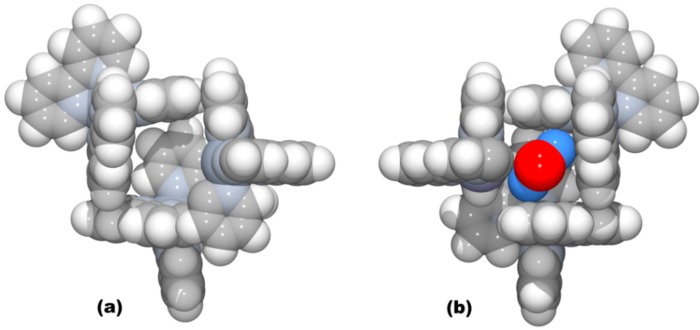
Space filling representation of cubic-shaped cages formed by three pairs of parallel-oriented bipyridine ligands proceeding from three adjacent [Zn^II^(bpy)_3_]^2+^ template cations: (**a**) view of the empty space of the cubic cage in Compound **1**; (**b**) view of the cubic cage in Compound **2** where the water molecule is located. Part of the bipyridine ligand located at the top of the cage is omitted for clarity.

However, in the actual case of the structure of **2**, the capture of water molecules into these cavities is expected, taking into account the aqueous preparation of the compound. Figure 5 shows the packing arrangement of three adjacent tris-chelated [Zn^II^(bpy)_3_]^2+^ cations exhibiting the cubic-shaped cavity, which is drawn with the frontal bpy ligand, partially omitted in order to have a free view into the cage where the H_2_O molecule is entrapped. The volume of this cubic cage in Compound **2** is about 45 Å^3^ (Figure S8). Consequently, the size decreasing of the [Zn^II^(bpy)_3_]^2+^ cationic template observed in **2** can be explained as a result of a steric pressure effect introduced by incorporation of additional water molecules into the cubic-shaped cavities.

### 3.2. Infrared Spectroscopy

The IR spectra of **1**–**8** are very similar (Figure S9), showing the characteristic absorption bands of the oxalate ligand in the regions 1610–1625 cm^−1^ (ν_as O–C–O_), 1305–1315 cm^−1^, 1350–1365 cm^−1^ (ν_s O–C–O_) and 795–805 cm^−1^ (δ _O–C–O_). The bands between 540 and 490 cm^−1^ are assigned to the Ru−O, Li/Na−O and M^II^−N stretching vibrations. The bands between 3100 and 2800 cm^−1^ and 1675–1400 cm^−1^ are attributed to the C–H, C_ar_–C_ar_ and C_ar_ = N stretching frequencies of the aromatic group. The series of bands at 1250–1000 cm^−1^ and near 3030–3050 cm^−1^ correspond to the aromatic =C–H stretching vibration.

### 3.3. Thermogravimetric Analysis

The thermal stability of **1**–**8** in air and nitrogen atmospheres was investigated. The thermogravimetric curves (TG and derivative TG), SDTA and mass spectrometry analysis of evacuated vapors for **1**–**8** in both air and nitrogen atmospheres are depicted in Figures S10–S11 and S13–S14 (Supplementary Materials), respectively. As represented, the thermogravimetric analysis results demonstrate similar decomposition behaviors, confirming the isomorphic nature of Compounds **1**–**8**. The degradation processes occurred in one single step simultaneously in both air and nitrogen atmospheres and very closely resemble each other. As summarized in Table S2 (Supplementary Materials), in air atmosphere, degradation of **1**–**8** proceeds through one continuous stage in which a mass loss of 63.6%–75.2% (depending on the compositional characteristics) is observed in the range 180–600 °C. This mass loss is associated with a broad exothermic peak on the SDTA and DSC curves (Figures S10–S12, Supplementary Materials) and corresponds to simultaneous decomposition of the organic template and ligand. The associated mass spectrometry *m/z* 18, 44 and 46 curves are in good agreement with the TG/dTG curves and occur as one broad maximum coinciding with the maximum of mass loss in dTG curves, suggesting continuous structure collapsing and oxidational degradation of the ligands.

Oppositely, in nitrogen atmosphere, the pyrolysis of Compounds **1**–**8** proceeds in three steps (Table S3, Supplementary Materials). As represented in Figures S13 and S14 (Supplementary Materials), these decomposition steps exhibit endothermic effects on the SDTA (Figures S13–S14, Supplementary Materials) and DSC curves (Figure S15; see the Supplementary Materials), which are associated with mass spectrometry *m/z* 15, 18 and 44 peaks, indicating stepwise decomposition of the polymeric architectures. Notably, the observed mass losses in nitrogen atmosphere do not correspond to those calculated theoretically (Table S3, Supplementary Materials). The found inconsistency between expected and theoretical mass losses can be attributed to the formation of carbon solid residues, which are the main product formed in the pyrolysis processes. Additionally, a composition of the residual solids of **1**–**8** after decomposition in air or nitrogen atmospheres was identified applying the powder X-ray diffraction technique. As a result, the residue powders formed after decomposition in air atmosphere consist of a mixture of RuO_2_, Li_2_O (for Compounds **2**, **4**, **6**, **8**) or Na_2_O (Compounds **1**, **3**, **5**, **7**) and M^II^O (M^II^ = Zn (**1**, **2**), Cu (**3**, **4**)) or M^IV^O_2_ (M^IV^ = Ru (**5**, **6**), Os (**7**, **8**)), while in nitrogen atmosphere, the residual composition has been identified as a mixture of Ru metal, Li_2_O (for Compounds **2**, **4**, **6**, **8**) or Na_2_O (Compounds **1**, **3**, **5**, **7**), M^II^O (M^II^ = Zn (**1**, **2**), Cu (**3**, **4**)) or metallic osmium (**7**, **8**).

### 3.4. UV–Vis Spectroscopy

**Figure 6:**
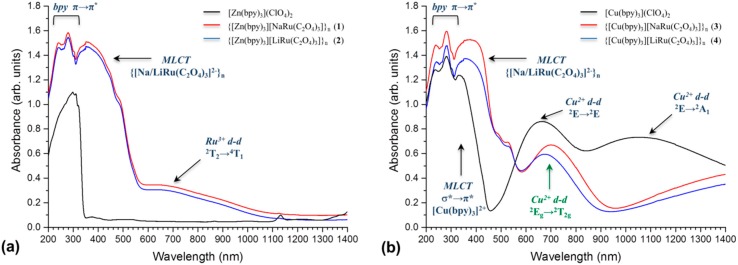

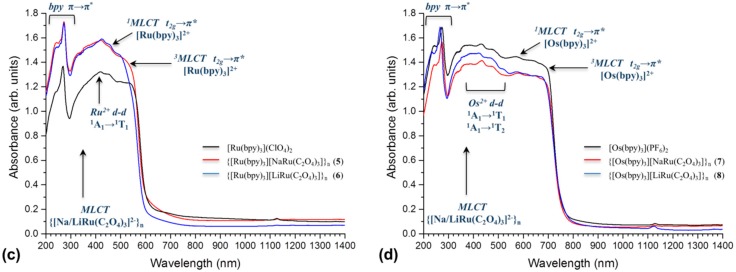
Comparison of room temperature UV–Vis–NIR diffuse-reflectance spectrum for: (**a**) {[Zn^II^(bpy)_3_][NaRu(C_2_O_4_)_3_]}_n_ (**1**), {[Zn^II^(bpy)_3_](H_2_O)[LiRu(C_2_O_4_)_3_]}_n_ (**2**) and [Zn(bpy)_3_](ClO_4_)_2_; (**b**) {[Cu^II^(bpy)_3_][M^I^Ru(C_2_O_4_)_3_]}_n_ and [Cu^II^(bpy)_3_](ClO_4_)_2_ (M^I^ = Na (**3**), Li (**4**)); (**c**) {[Ru^II^(bpy)_3_][M^I^Ru(C_2_O_4_)_3_]}_n_ and [Ru(bpy)_3_](ClO_4_)_2_ (M^I^ = Na (**5**), Li (**6**)); (**d**) {[Os^II^(bpy)_3_][M^I^Ru(C_2_O_4_)_3_]}_n_ and [Os(bpy)_3_](PF_6_)_2_ (M^I^ = Na (**7**), Li (**8**)).

The room-temperature UV–Vis–NIR diffuse reflectance spectra of the powder samples corresponding to **1**–**8** are represented in Figure 6. All spectra consist of three groups of bands: the high energy bands observed between 200 and 330 nm are assigned to the π→π* transition of bpy ligands; the intense broad band at *ca*. 400 nm corresponds to the MLCT transition in [Na/LiRu(C_2_O_4_)_3_]^2−^ units; whereas the weaker bands in the VIS-NIR region have been assigned to ligand-field transitions within the [Z^II^(bpy)_3_]^2+^ cationic templates of Compounds **1**–**8**.

Figure 6a shows a comparison of the diffuse-reflectance spectra of **1**, **2** and [Zn(bpy)_3_](ClO_4_)_2_ compounds. As expected, the spectrum of the [Zn(bpy)_3_]^2+^ complex does not appear to have *d*–*d* transitions due to the close shell electronic configuration (t_2g_^6^e_g_^4^) for the *d*^10^ Zn^2+^ ion [68]. However, the spectra of **1** and **2** exhibit a broad adsorption band *ca*. 700 nm, which was assigned to the *d*–*d* (Ru^3+^) spin-forbidden ^2^T_2_→^4^T_2_ transition within the [Na/LiRu(C_2_O_4_)_3_]^2−^ framework units [94]. The Vis–NIR spectral region of **3** and **4** coordination polymers templated by the [Cu(bpy)_3_]^2+^ cationic complex (Figure 6b) reveal the adsorption band of *ca*. 690 nm that was assigned to the ^2^E_g_→^2^T_2g_ single electron transition, which is expected in the octahedral crystal field for the Cu^2+^ ion (^2^T_2g_) with the t_2g_^5^e_g_^4^ excited electronic state [95].

Normally, the octahedral coordination of Cu^2+^ ions undergoes Jahn–Teller distortion, leading to the trigonally-distorted pseudo-*D*_3_ symmetry and can be observed in the corresponding spectrum of the [Cu(bpy)_3_](ClO_4_)_2_ compound (Figure 6b), where *d*–*d* transitions appeared as a medium-strong band of *ca*. 680 nm, and a sharp band of *ca*. 1100 nm should be treated as the trigonal field and assigned to ^2^E→^2^E and ^2^E→^2^A_1_ transitions, respectively [96]. Based on these observations, the fact that the {[M^I^Ru(C_2_O_4_)_3_]^2−^}_n_ (M^I^ = Na^+^, Li^+^) anionic framework rigidly restricts Jahn–Teller distortion in the guest [Cu(bpy)_3_]^2+^ cationic complex is concluded. Furthermore, the corresponding structural distortion parameters (bond angle variance (σ^2^) and mean quadratic elongation (λ)) calculated for the [Cu(bpy)_3_]^2+^ complex in **3** and **4** frameworks, which are summarized in Table 1, suggest that the coordination environment of the Cu^2+^ ion in the template cationic complex exhibits more regularized octahedral geometry than that found for the corresponding free salt form.

The diffuse-reflectance spectra of Compounds **5** and **6** are similar with respect to the corresponding [Ru(bpy)_3_](ClO_4_)_2_ complex, and the Vis–NIR region consists of several high intensity bands (Figure 6c), which are attributed to electron transitions within the low-spin [Ru(bpy)_3_]^2+^ complex, where the Ru^2+^ ion possesses the t_2g_^5^e_g_^1^ electronic configuration [95]. Thus, the absorption band of *ca*. 450 nm is assigned to the ^1^A_1_→^1^T_1_ transition. Moreover, the shoulder centered at 480 nm corresponds to the *t*_2g_→π* metal-ligand charge transfer (^1^MLCT) transition, while the broad shoulder observed at 560 nm belongs to a spin-forbidden third *t*_2g_→π* metal-ligand charge transfer (^3^MLCT) transition [97]. Similarly, Compounds **7** and **8** exhibit diffuse-reflectance spectra close to the [Os(bpy)_3_](PF_6_)_2_ complex. As shown in Figure 6d, the Vis–NIR region of spectra consists of several overlapped bands located from 410 –520 nm and was assigned to the ^1^A_1_→^1^T_2_ and ^1^A_1_→^1^T_1_ *d*‒*d* transitions, which are expected for the low-spin [Os(bpy)_3_]^2+^ complex with the Os^2+^ ion in the t_2g_^5^e_g_^1^ ground state [95]. Similarly to [Ru(bpy)_3_]^2+^-contained compounds, the diffuse-reflectance spectra of **7** and **8**, as well as [Os(bpy)_3_](PF_6_)_2_ exhibit characteristic shoulders localized from 560 800 nm, which are attributed to the *t*_2g_→π* metal-ligand charge transfer (MLCT) along with the spin-forbidden third *t*_2g_→π* metal-ligand charge transfer (^3^MLCT) transition [98].

The band gaps of **1**–**8** were estimated from Tauc plots [99] obtained from UV–Vis diffuse-reflectance data transformed by the Kubelka–Munk function (Figure S16). The band gaps (*E*_g_) were determined extrapolating the intersection point between the baseline and the linear portion of the adsorption edge in a plot represented as function (*αhυ*)^3/2^ against energy (*hυ,* eV). The optical adsorption related to *E*_g_ in the region of MLCT transition, which is assumed to be directly forbidden, can be assessed at 2.54 eV for **1**, 2.31 eV for **2**, 2.68 eV for **3**, 2.67 eV for **4**, 2.10 eV for **5**, 2.11 eV for **6**, 1.68 eV for **7** and 1.67 eV for **8**, respectively. The determined values of band gaps for coordination polymers **1**–**8** follow the order **3** ≈ **4** > **1** > **2** > **5** ≈ **6** > **7** ≈ **8**.

The efficiency of photoinduced energy and electron migration processes occurring between the photosensitive component and the catalytically-active centers in MOFs/CPs upon light irradiation are essential goals in the rational design of photo-catalytically-active MOFs/CPs [100]. Thus, inspired by the early study of Kimura *et al.* [101], which demonstrated efficient intramolecular energy and electron transfer taking place in a homogeneous solution between [Co(C_2_O_4_)_3_]^3−^ and [Ru(bpy)_3_]^2+^ complexes, and supported by later works of Decurtins *et al.* [62,63,69,70,74,76], which evidenced the existence of *hv*-assisted resonant energy migration between [Cr(C_2_O_4_)_3_]^3−^ and [M^II/III^(bpy)_3_]^2+/3+^ components in {[Z^II/III^(bpy)_3_][NaCr(C_2_O_4_)_3_]}_n_ (Z^II^ = Ru^2+^, Zn^2+^, Os^2+^, Fe^2+^; M^III^ = Rh^3+^, Cr^3+^) coordination networks, we envisioned that the coordination polymers **1**–**8** can act as self-supported photocatalysts.

### 3.5. Photocatalytic Activity

**Figure 7:**
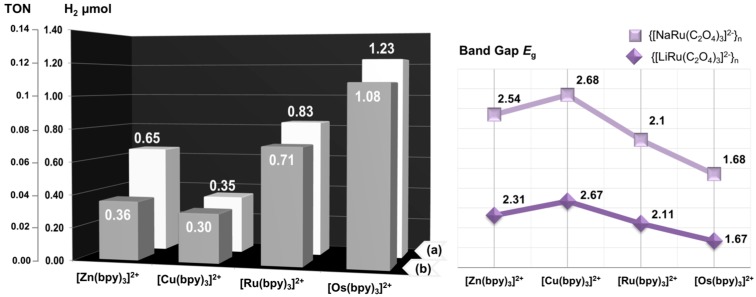
(**Left**) Amounts and TON values corresponding to H_2_ evolved during the reductive water splitting reaction using photocatalysts **1**–**8**, which contain [Zn(bpy)_3_]^2+^, [Cu(bpy)_3_]^2+^, [Ru(bpy)_3_]^2+^ and [Os(bpy)_3_]^2+^ complex cations incorporated in (**a**) {[LiRu(C_2_O_4_)_3_]^2−^}_n_ and (**b**) {[NaRu(C_2_O_4_)_3_]^2−^}_n_ networks under 8 h of UV (≤366 nm) light irradiation. Photoreaction mixtures contained 10 µmol of heterogeneous catalyst, 7 mL of TEA and 10 mL of H_2_O. TON = μmol of H_2_ evolved/10 μmol of catalyst. (**Right**) The values of band gaps were determined by diffuse-reflectance measurements for Compounds **1**–**8**.

The photocatalytic splitting of water for hydrogen production using Compounds **1**–**8** under UV (≤366 nm) and VIS (≥417 nm) light irradiation was examined. In a typical experiment, the reactions were performed in a reactor equipped with a refrigerated 500-W Hg-lamp (≤366 nm) and using 10 μmol of heterogeneous catalyst **1**–**8** dispersed in a water (H_2_O)/triethylamine (TEA) mixture (*v*/*v* = 1.4:1), where TEA acts as the electron donor. The amounts of H_2_ produced over **1**–**8** photocatalysts under 8 h of UV light irradiation are depicted in Figure 7.

As seen in Figure 7 (left), the heterogeneous catalysts **1**–**8** are active in the photoreductive water splitting reaction, forming 1.26 μmol (TON of 0.12) of H_2_ after 8 h under UV light irradiation. Catalysts **7** and **8** exhibit the highest photocatalytic performance, compared to the activity of the other compounds, and their activities decrease through the sequence **8** > **7** > **6** > **5** > **2** > **1** ≈ **4** > **3**. Interestingly, this sequence of photocatalytic activity is directly opposed to the calculated band gaps for these compounds (Figure 7, right). Therefore, the synergistic effects of the smallest band gap and chemical nature of the [Z^II^(bpy)_3_]^2+^ cationic template are the main factors determining the photocatalytic activities of **1**–**8** under UV light irradiation. Blank reactions were performed to ensure that H_2_ production was light-promoted and conducted over a heterogeneous catalyst. One blank was UV-illuminated without the catalyst, and another was in the dark with the catalyst under the same experimental conditions. No H_2_ was detected in the above two blank tests. A “hot filtration” test was conducted with **6**, in which the heterogeneous catalyst, previously exposed to 8 h of reaction under UV light, was removed by centrifugation, and the transparent uncolored reactant solution was returned into the photolysis cell (previously degassed and filled with Ar) for an additional consecutive photocatalytic run. As a result, no H_2_ was detected, which indicates that the detected photoactivities are promoted by heterogeneous catalysts rather than by leached soluble species.

**Figure 8:**
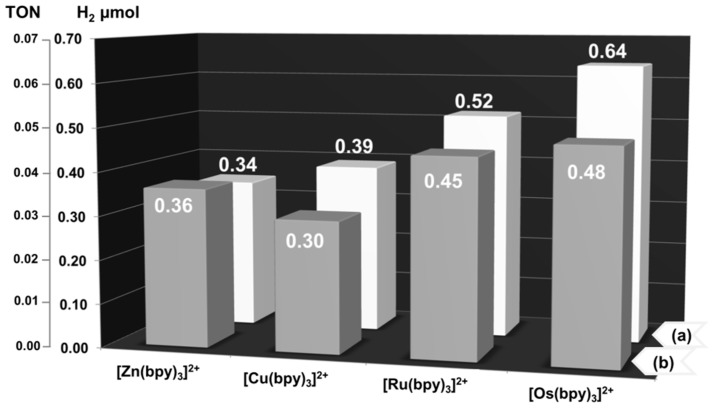
Amounts and TON values corresponding to H_2_ evolved during the reductive water splitting reaction using photocatalysts **1**–**8**, which contain [Zn(bpy)_3_]^2+^, [Cu(bpy)_3_]^2+^, [Ru(bpy)_3_]^2+^ and [Os(bpy)_3_]^2+^ complex cations incorporated in (a) {[LiRu(C_2_O_4_)_3_]^2−^}_n_ and (b) {[NaRu(C_2_O_4_)_3_]^2−^}_n_ networks under 8 h of Vis (≥417 nm) light irradiation (left). Photoreaction mixtures contained 10 µmol of heterogeneous catalyst, 7 mL of TEA and 10 mL of H_2_O. TON = μmol of H_2_ produced/10 μmol of catalyst.

Additionally, the photocatalytic activities of **1**–**8** were examined under VIS light irradiation under the same reaction conditions. As shown in Figure 8, all coordination compounds also catalyze the photoreduction of water to H_2_, albeit less efficiently. These differences in photocatalytic activities of **1**–**8** under UV and VIS irradiation can be explained in light of the distinct mechanisms of energy transfer taking place within the {[Z^II/III^(bpy)_3_][M^I^M^III^(C_2_O_4_)_3_]}_n_ host-guest system, which earlier was evidenced by Decurtins *et al.* for analogous {[Z^II/III^(bpy)_3_][NaCr(C_2_O_4_)_3_]}_n_ (Z^II^ = Ru^2+^, Zn^2+^, Os^2+^, Fe^2+^; Z^III^ = Rh^3+^, Cr^3+^) compounds [70,73,102]_._

**Table 2:** Comparison of photocatalytic performances of known MOFs/CPs used in the photoreduction of water to H_2_ under UV and Vis light.

MOF/CP	H_2_ (μmol)	T (h)	TON	TOF (TON·h^−1^)	λ (nm)	Ref.
[Ru^II,III^_2_(*p*-BDC)_2_BF_4_]_n_	29.3	4	47.0 ^1^	11.7	>420	[47]
[Ru^II,III^_2_(*p*-BDC)_2_Cl]_n_ [Ru^II,III^_2_(p-BDC)_2_Br]_n_	13.5 46.7	4	5.38 ^2^ 18.7 ^2^	1.34 4.67	>420	[48]
Ti–MOF–NH_2_@Pt	11.7	3	1.17 ^2^	0.39	>420	[49]
Ti–MOF–NH_2_@Pt	15.5	3	1.5 ^2^	0.5	>420	[50]
NH_2_–UiO–66(Zr) NH_2_–UiO–66(Zr)@Pt	107.1 125	3	2.38 ^2^ 2.77 ^2^	0.93 0.79	370	[51]
MIL–101(Cr)@CdS/Pt	300	1	150 ^2^	75.5	>420	[52]
UiO–66(Zr)@Pt UiO–66(Zr)@RhB UiO–66(Zr)@RhB/Pt	0.97 0.67 1.4	5	19.5 ^3^ 13.5 ^3^ 28 ^3^	3.9 2.7 5.6	>420	[53]
UiO–67[Ir(ppy)_2_(bpy)]@Pt	64.8	6	1620 ^1^	270	420	[54]
MOF–253–Pt	3000	34	5.6 ^1^	0.17	420	[55]
UiO–66–[FeFe](dcbdt)(CO)_6_	3.5	2.5	0.7 ^2^	0.28	470	[56]
{[Sm_2_Cu_5_(OH)_2_(pydc)_6_(H_2_O)_8_]·I_8_} {[Eu_2_Cu_5_(OH)_2_(pydc)_6_(H_2_O)_8_]·I_8_} {[Gd_2_Cu_5_(OH)_2_(pydc)_6_(H_2_O)_8_]·I_8_} {[Tb_2_Cu_5_(OH)_2_(pydc)_6_(H_2_O)_8_]·I_8_}	979.0 1131.4 1025.2 1052.5	5	9790 ^3^ 11,314 ^3^ 10,252 ^3^ 10,525 ^3^	1958.0 2262.8 2050.4 2105.0	420	[57]
(TBA)_2_[Cu^II^(BBTZ)_2_(*x*-Mo_8_O_26_)]	4.68	6	0.05 ^2^	0.008	<400	[58]
{[AlOH)]_2_H_2_TCPP(DMF_3_–(H_2_O)_2_}	3.15	8	900 ^3^	112.5	420	[59]
{[Zn(bpy)_3_][NaRu(C_2_O_4_)_3_]}_n_	0.36	8	0.04 ^1,3^ 36 ^2^	0.005 4.5	≥417	This work
	0.36		0.04 ^1,3^ 36 ^2^	0.005 4.5	≤366	
{[Zn(bpy)_3_][LiRu(C_2_O_4_)_3_]}_n_	0.34		0.03 ^1,3^ 34 ^2^	0.004 4.25	≥417	
	0.65		0.06 ^1,3^ 65 ^2^	0.007 8.12	≤366	
{[Cu(bpy)_3_][NaRu(C_2_O_4_)_3_]}_n_	0.30		0.03 ^1,3^ 30 ^2^	0.004 3.75	≥417	
	0.30		0.03 ^1,3^ 30 ^2^	0.004 3.75	≤366	
{[Cu(bpy)_3_][LiRu(C_2_O_4_)_3_]}_n_	0.39		0.04 ^1,3^ 39 ^2^	0.005 4.87	≥417	
	0.35		0.04 ^1,3^ 35 ^2^	0.004 4.37	≤366	
{[Ru(bpy)_3_][NaRu(C_2_O_4_)_3_]}_n_	0.45		0.04 ^1,3^ 45 ^2^	0.005 5.62	≥417	
	0.71		0.07 ^1,3^ 71 ^2^	0.009 8.75	≤366	
{[Ru(bpy)_3_][LiRu(C_2_O_4_)_3_]}_n_	0.52		0.05 ^1,3^ 52 ^2^	0.006 6.5	≥417	
	0.83		0.08 ^1,3^ 83 ^2^	0.01 10.4	≤366	
{[Os(bpy)_3_][NaRu(C_2_O_4_)_3_]}_n_	0.48		0.05 ^1,3^ 48 ^2^	0.006 6	≥417	
	1.08		0.11 ^1,3^ 108 ^2^	0.13 13.5	≤366	
{[Os(bpy)_3_][LiRu(C_2_O_4_)_3_]}_n_	0.64		0.06 ^1,3^ 64 ^2^	0.008 8	≥417	
	1.23		0.12 ^1,3^ 123 ^2^	0.15 15.4	≤366	

^1^ TON = μmol H_2_·μmol ^−1^ MOF; ^2^ TON = μmol H_2_·mg^−1^ MOF; ^3^ TON= μmol H_2_·g^−1^ MOF.

According to the proposed mechanism, the resonant energy migration takes place between the [M^III^(C_2_O_4_)_3_]^3−^ and [Z^II/III^(bpy)_3_]^2+/3+^ components of {[Z^II/III^(bpy)_3_][M^I^M^III^(C_2_O_4_)_3_]}_n_, in which energy transfer to the [Z^II/III^(bpy)_3_]^2+/3+^ component is more efficient that to [M^III^(C_2_O_4_)_3_]^3−^. Evidently, the UV region of adsorption of **1**–**8** consists of the bands attributed to MLCT transition within the {[M^I^Ru(C_2_O_4_)_3_]^3−^}_n_ network, which indicates that under UV-light, the [Ru(C_2_O_4_)_3_]^3−^ unit behaves as the photosensitive component, efficiently transferring the energy received upon excitation to [Z^II^(bpy)_3_]^2+^ guests (Z^II^ = Zn^2+^, Cu^2+^, Ru^2+^, Os^2+^), leading to better photocatalytic performances.

Meanwhile, the VIS region of **1**–**8** consists of absorption bands attributed to MLCT transitions within the [Z^II^(bpy)_3_]^2+^ component of the coordination polymers, which suggests that tris-bipyridine guests are photosensitive components towards VIS light. Besides the low rate of energy transfer efficiency from the [Z^II^(bpy)_3_]^2+^ to [Ru(C_2_O_4_)_3_]^3−^ components of the **1**–**8** frameworks, this leads to the decreasing of the photocatalytic performance in the water-splitting reaction.

Taking into account the above-mentioned statements, we can propose that the reaction includes the following steps: promotion of the [Ru^III^(C_2_O_4_)_3_]^3−^ structural unit of the framework to its excited state under UV light irradiation; resonant energy migration from the exited ([Ru^III^(C_2_O_4_)_3_]^3−^)* unit to [Z^II^(bpy)_3_]^2+^ cationic guest through Forster and Dexter energy transfer mechanisms (see the additional references in the Supplementary Materials), causing the latter to go into the exited state; the exited species ([Z^II^(bpy)_3_]^2+^)* transfers an electron, located on one bpy ligand, to the water proton and returns to its initial state through the oxidation of a sacrificial reductant TEA.

In order to confirm the recyclability of photocatalysts, the photocatalytic reaction of reductive water splitting was repeated four times with Compound **6**, where after each catalytic cycle, the heterogeneous solid was separated by centrifugation, washed several times with distillated water and reused in the next consecutive photocatalytic run. As shown in Figure S17, the amounts of H_2_ evolved after 8 h of UV light irradiation in each consecutive photocatalytic cycle decrease slightly, probably due to the loss of catalyst upon recycling manipulation procedures. Moreover, the closely similar photocatalytic activities of recycled catalyst suggest that Compound **6** does not undergo photodecomposition or deactivation, at least after four repeated catalytic runs. Additionally, to confirm the stability of heterogeneous catalyst, after each recycling run, the reused material **6** was checked by XRD, and as evidenced from the comparison of those diffractograms (Figure S18), photocatalyst **6** maintains its crystallinity and structural integrity during the water splitting reaction. These results indicate that coordination polymers **1**–**8** behave as stable, active and reusable heterogeneous catalysts for the photoreductive water-splitting reaction. Moreover, we compare the photocatalytic activities of **1**–**8** with other known MOFs and CPs able to photo-split water to H_2_ (Table 2).

The presented results reveal that coordination polymers **1**–**8** show moderate photocatalytic activity towards H_2_ generation under VIS light compared to known MOFs/CPs; meanwhile, under UV light, they exhibit higher photocatalytic efficiencies. It is reasonable to conclude that {M^I^Ru(C_2_O_4_)_3_]^2−^}_n_ (M^I^ = Na, Li) anionic frameworks selectively templated by [Z^II^(bpy)_3_]^2+^ (Z^II^ = Zn^2+^, Cu^2+^, Ru^2+^, Os^2+^) cationic complexes can be viewed as designable and efficient heterogeneous catalysts for UV light-promoted photoreactions.

## 4. Conclusions

A series of [Z^II^(bpy)_3_]^2+^-templated (Z^II^ = Zn^2+^ (**1**, **2**); Cu^2+^ (**3**, **4**); Ru^2+^ (**5**, **6**); Os^2+^ (**7**, **8**)) and {[M^I^Ru(C_2_O_4_)_3_]^2−^}_n_ (M^I^ = Na^+^, Li^+^) anionic frameworks were obtained through self-assembly at room temperature in aqueous media. The anionic framework structures of **1**–**8** consist of triangular cages, which selectively and homogeneously encapsulate [Z^II^(bpy)_3_]^2+^ complex cations. Furthermore, the electronic configuration of the cationic guest complexes is shown to be influenced by the framework. In addition, the [Z^II^(bpy)_3_]^2+^ templates embedded within the anionic cages of {[M^I^Ru(C_2_O_4_)_3_]^2−^}_n_ (M^I^ = Na^+^, Li^+^) networks undergo a 6.9%–14.4% expansion as a result of the electrostatic interaction between them. The MLCT band gaps in **1**−**8** can easily be tuned by the [Z^II^(bpy)_3_]^2+^ cationic guest and, as has been shown, follow the order of **3** ≈ **4** > **1** > **2** > **5**~**6** > **7** ≈ **8**. The **1**–**8** CPs exhibit catalytic activity in UV light-promoted H_2_ evolution from water, reaching a total TON of 123, where photocatalytic efficiencies follow the order **8** > **7** > **6** > **5** > **2** > **1** ≈ **4** > **3**. Under VIS light irradiation, the CPs **1**–**8** exhibit moderate photocatalytic activities, as compared to known MOFs/CPs, with an enhanced catalytic rate following the order of **8** > **6** > **7** > **5** > **4** > **1** ≈ **2** > **3**, leading to the production of H_2_ with a total TON of 64. Moreover, heterogeneous catalysts remain active for four consecutive usages and preserve the structural integrity and crystallinity.

These results highlight that rational synthesis of 3D anionic architectures using a target cationic guest, such as [Z^II^(bpy)_3_]^2+^, provides a powerful route for the construction of multifunctional guest-encapsulated CPs with a predictable structural topology and desirable properties.

## Supplementary Materials

The supplementary materials are available online at www.mdpi.com/2073-4360/8/2/48/s1.

## Abbreviations

The following abbreviations are used in this manuscript:

### Abbreviations

CPs: Coordination polymers
MOFs: Metal-organic frameworks
BDC: Benzene-1,4-dicarboxylate
bpy: 2,2’-Bipyridine
EDTA: Ethylenediaminetetraacetate
MV^2+^: *N*,*N*’-Dimethyl-4,4’-bipyridinium
ppy: 2-Phenylpyridine
dcbdt: 1,4-Dicarboxylbenzene-2,3-dithiolate
TBA: Tetrabutylammonium cation
BBTZ: 1,4-bis(1,2,4-Triazol-1-ylmethyl)-benzene
H_2_TCPP: Tetra(4-carboxyl-phenyl)porphyrin
SEM: Scanning electron microscopy
TGA: Thermogravimetric analysis
XRD: X-ray diffraction
TEA: Triethylamine
3D: Three-dimensional
SBU: Secondary building unit
SDTA: Simultaneous difference thermal analysis
DSC: Differential scanning calorimetry
MLCT: Metal-to-ligand charge transfer
