# Supplementary material for: Tris(bipyridine)Metal(II)-Templated Assemblies of 3D Alkali-Ruthenium Oxalate Coordination Frameworks: Crystal Structures, Characterization and Photocatalytic Activity in Water Reduction
Source: Polymers (Basel). 2016 Feb 15;8(2):48. doi: 10.3390/polym8020048 (PMC6432594; doi:10.3390/polym8020048)
Supplement: Supplementary file 1 [file polymers-08-00048-s001.pdf]

Supplementary Materials: Tris(bipyridine)Metal(II)-Templated Assemblies of 3D Alkali-Ruthenium Oxalate Coordination Frameworks: Crystal Structures, Characterization and Photocatalytic Activity in Water Reduction

Alla Dikhtiarenko, Pedro Villanueva-Delgado, Rafael Valiente, José R. García and José Gimeno

Table S1. Crystallographic data and structure refinements for compounds **1**–**8**.

Compound	1	2	3
Formula	$C_{36}H_{24}N_6NaO_{12}RuZn$	$C_{36}H_{26}LiN_6O_{13}RuZn$	$C_{36}H_{24}CuN_6NaO_{12}Ru$
FW (g mol^{-1})	922.06	924.03	920.22
T (K)	293(2)	293(2)	298(1)
Wavelength (Å)	1.54184	0.71073	1.54184
Crystal system	Cubic	Cubic	Cubic
Space group	$P2_13$	$P2_13$	$P2_13$
a (Å)	15.7549(2)	15.3337(4)	15.6236(2)
α (°)	90.0	90.0	90.0
V (Å^3)	3,910.70(8)	3,605.3(3)	3,813.67(8)
Z	4	4	4
D_{calcd} (g/cm^3)	1.566	1.699	1.603
F(000)	1,852	1,852	1,848
μ (mm^{-1})	4.589	1.160	4.602
Crystal size (mm^3)	0.21 × 0.16 × 0.14	0.61 × 0.49 × 0.22	0.15 × 0.08 × 0.03
θ_{range} data collect. (°)	4.0 to 70.3	3.8 to 31.2	4.0 to 70.4
Index ranges	$-17 \le h \le 14$, $-11 \le k \le 11$, $-15 \le l \le 19$	$-15 \le h \le 15$, $-21 \le k \le 10$, $-4 \le l \le 20$	$-17 \le h \le 17$, $-18 \le k \le 13$, $-18 \le l \le 10$
Reflection collected	4,945	7,387	7,070
Independent reflections	2,371[R_{int} = 0.082]	3,525[R_{int} = 0.034]	2,396[R_{int} = 0.065]
T_{max} and T_{min}	1 and 0.802	0.837 and 0.626	0.883 and 0.672
Data/restraints/paramets.	2,371/0/173	3,525/0/176	2,396/0/172
Final R indices [$I > 2\sigma(I)$]	R_1 = 0.074, wR_2 = 0.211	R_1 = 0.052, wR_2 = 0.144	R_1 = 0.039, wR_2 = 0.078
R indices (all data)	R_1 = 0.092, wR_2 = 0.235	R_1 = 0.055, wR_2 = 0.146	R_1 = 0.061, wR_2 = 0.085
Flack parameter	0.43(3)	0.15(3)	0.007(14)
GOF on F^2	1.082	1.17	1.002

Table S1. *Cont.*

Compound	4	5	6
Formula	$C_{36}H_{24}CuLiN_6O_{12}Ru$	$C_{36}H_{24}N_6NaO_{12}Ru_2$	$C_{36}H_{24}LiN_6O_{12}Ru_2$
FW (g mol^{-1})	957.74	941.69	1,046.9
T (K)	293(2)	293(2)	293(2)
Wavelength (Å)	0.71073	1.54184	1.54184
Crystal system	Cubic	Cubic	Cubic
Space group	$P2_13$	$P2_13$	$P2_13$
a (Å)	15.3725(1)	15.5466(1)	15.3785(1)
α (°)	90.0	90.0	90.0
V (Å^3)	3,632.73(6)	3,757.56(4)	3,636.99(4)
Z	4	4	4
D_{calcd} (g/cm^3)	1.653	1.693	1.720
F(000)	1,816	1,908	1,876
μ (mm^{-1})	1.075	7.247	7.363
Crystal size (mm^3)	0.29 × 0.26 × 0.16	0.14 × 0.12 × 0.08	0.26 × 0.25 × 0.14
θ_{range} data collect. (°)	3.2 to 31.3	4.0 to 70.4	4.1 to 70.5
Index ranges	$-6 \le h \le 22$, $-17 \le k \le 13$, $-11 \le l \le 21$	$-12 \le h \le 12$, $0 \le k \le 13$, $1 \le l \le 18$	$-12 \le h \le 12$, $0 \le k \le 13$, $1 \le l \le 18$
Reflection collected	7,220	7,080	9,446
Independent reflections	3,586[R_{int} = 0.028]	2,367[R_{int} = 0.049]	2,335[R_{int} = 0.040]
T_{max} and T_{min}	1 and 0.957	0.625 and 0.471	0.389 and 0.320
Data/restraints/paramets	3,586/0/172	2,367/0/172	2,335/0/172
Final *R* indices [$I > 2\sigma(I)$]	R_1 = 0.039, wR_2 = 0.108	R_1 = 0.029, wR_2 = 0.074	R_1 = 0.025, wR_2 = 0.068
R indices (all data)	R_1 = 0.046, wR_2 = 0.112	R_1 = 0.034, wR_2 = 0.077	R_1 = 0.025, wR_2 = 0.068
Flack parameter	−0.006(18)	−0.007(14)	0.021(12)
GOF on F^2	1.105	1.031	1.101

Table S1. *Cont.*

Compound	7	8
Formula	$C_{36}H_{24}N_6NaO_{12}OsRu$	$C_{36}H_{24}LiN_6O_{12}OsRu$
FW (g mol^{-1})	1,046.9	1,030.85
T (K)	293(2)	293(2)
Wavelength (Å)	1.54184	1.54184
Crystal system	Cubic	Cubic
Space group	$P2_13$	$P2_13$
a (Å)	15.5139(1)	15.3442(6)
α (°)	90.0	90.0
V (Å^3)	3,733.90(4)	3,612.75(8)
Z	4	4
D_{calcd} (g/cm^3)	1.862	1.895
F(000)	2,036	2,004
μ (mm^{-1})	10.346	10.567
Crystal size (mm^3)	0.08 × 0.06 × 0.03	0.07 × 0.03 × 0.02
θ_{range} data collect. (°)	4.0 to 70.4	4.1 to 70.7
Index ranges	$-17 \le h \le 17$, $-8 \le k \le 18$, $-13 \le l \le 10$	$-18 \le h \le 8$, $-12 \le k \le 18$, $-15 \le l \le 18$
Reflection collected	4,956	2,302
Independent reflections	2,323[R_{int} = 0.046]	2,302[R_{int} = 0.053]
T_{max} and T_{min}	0.081 and 0.064	0.817 and 0.699
Data/restraints/paramets.	2,323/0/172	2,302/0/172
Final R indices [$I > 2\sigma(I)$]	R_1 = 0.035, wR_2 = 0.088	R_1 = 0.029, wR_2 = 0.074
R indices (all data)	R_1 = 0.042, wR_2 = 0.091	R_1 = 0.034, wR_2 = 0.077
Flack parameter	−0.03(2)	0.00(2)
GOF on F^2	1.037	1.057

Table S2. Summary of thermogravimetric data in air atmosphere for compounds **1–8**.

Com-d	Molecular formula	FW	T_{range}, °C	T_{max}, °C	Weight loss, % Obs./Calc.	*m*/z	Enthalpy, J/g	Residue [1]
1	$C_{36}H_{24}N_6NaO_{12}RuZn$	922.1	240/420	295	73.0/73.4	H_2O (18), CO_2 (44), NO_2 (46)	−8,839	RuO_2, ZnO, Na_2O
2	$C_{36}H_{26}LiN_6O_{13}RuZn$	923.9	180/420	290	75.2/75.4		−12,100	RuO_2, ZnO, Li_2O
3	$C_{36}CuH_{24}N_6NaO_{12}Ru$	920.2	200/400	270	73.7/73.5		−10,930	RuO_2, CuO, Na_2O
4	$C_{36}CuH_{24}LiN_6O_{12}Ru$	904.2	200/380	275	75.0/74.8		−12,050	RuO_2, CuO, Li_2O
5	$C_{36}H_{24}N_6NaO_{12}Ru_2$	957.7	240/440	300	69.0/69.6		−15,920	RuO_2, Na_2O
6	$C_{36}H_{24}LiN_6O_{12}Ru_2$	941.7	240/400	300	70.4/70.2		−14,770	RuO_2, Li_2O
7	$C_{36}H_{24}N_6NaO_{12}OsRu$	1,046.9	220/460	300	63.6/63.8		−13,140	RuO_2, OsO_2, Na_2O
8	$C_{36}H_{24}LiN_6O_{12}OsRu$	1,030.8	230/600	310	64.7/64.1		−14,590	RuO_2, OsO_2, Li_2O

[1] The residue composition was confirmed by EDX analysis and powder X-ray diffraction.

Table S3. Summary of thermogravimetric data in nitrogen atmosphere for compounds **1–8**.

Com-d	Molecular formula	FW	T_{range}, °C	T_{max}, °C	Weight loss, % Obs.[1]/Calc.	m/z	Enthalpy, J/g	Residue [2]
1	$C_{36}H_{24}N_6NaO_{12}RuZn$	922.1	260/360<br>360/430<br>440/550	330<br>400<br>520	59.0/76.9	NH_3 (15), H_2O (18), CO_2 (44)	451<br>261	Ru, ZnO, Na_2O
2	$C_{36}H_{26}LiN_6O_{13}RuZn$	923.9	200/380<br>380/440<br>460/580	360<br>400<br>520	58.0/78.6		480<br>340	Ru, ZnO, Li_2O
3	$C_{36}CuH_{24}N_6NaO_{12}Ru$	920.2	220/280<br>300/380<br>400/600	250<br>330<br>560	61.1/77.0		81<br>87<br>226	Ru, CuO, Na_2O
4	$C_{36}CuH_{24}LiN_6O_{12}Ru$	904.2	200/280<br>280/380<br>540/600	250<br>320<br>570	57.6/78.4		53<br>117<br>257	Ru, CuO, Li_2O
5	$C_{36}H_{24}N_6NaO_{12}Ru_2$	957.7	300/400<br>440/540<br>560/700	360<br>510<br>600	45.5/75.7		361<br>130	Ru, Na_2O
6	$C_{36}H_{24}LiN_6O_{12}Ru_2$	941.7	320/420<br>450/560<br>560/700	380<br>520<br>600	43.9/76.9		430<br>109	Ru, Li_2O
7	$C_{36}H_{24}N_6NaO_{12}OsRu$	1046.9	300/420<br>460/550<br>560/740	350<br>520<br>640	37.8/69.2		325<br>50	Ru, Os, Na_2O
8	$C_{36}H_{24}LiN_6O_{12}OsRu$	1030.8	260/400<br>460/580<br>580/720	340<br>560<br>650	39.7/70.3		295<br>147	Ru, Os, Li_2O

[1] The difference between observed and calculated mass losses in nitrogen atmosphere due to formation of carbon solid residues as a product of partial oxidation of organic ligands; [2] The residue composition was confirmed by EDX analysis and powder X-ray diffraction.

Band Gap Calculations

The band gap energies of **1**–**8** were calculated by Tauc plots of diffuse–reflectance data. According to equation:

$$\alpha h\upsilon = A(h\upsilon - E_g)^{1/n}$$

where α, υ, A and E_g are the absorption coefficient, light frequency, proportionality constant, and band gap, respectively. In the equation, n can take on values of 3, 2, 3/2 or 1/2, corresponding to indirect (forbidden), indirect (allowed), direct (forbidden), and direct (allowed) transitions, respectively. The plots of $(\alpha h\upsilon)^n$ versus photon energy $h\upsilon$, so-called Tauc plots, yield the value of the band gap when extrapolated to the baseline. In case of diffuse-reflectance measurements ($I = R$), the Kubelka-Munk radiative transfer model can be employed to extract α:

$$f(R) = \frac{(1-R)^2}{2R} = \frac{\alpha}{s}$$

where $f(R)$ is the Kubelka-Munk function and s is the scattering coefficient. Assuming that s is wavelength independent, the $f(R)$ is proportional to α and the Tauc plots can be represented using $f(R)$ in place of α, as follow equation:

$$(f(R)h\upsilon)^n = A(h\upsilon - E_g)$$

Thus, values of band gaps for coordination polymers **1**–**8** were determined as extrapolation to energy axis of point of intersection between baseline and linear fraction of $(f(R)h\upsilon)^{3/2}$ (where n = 3/2 was used as a coefficient characterized direct forbidden MLCT transition).

Photocatalytic mechanism and energy transfer in Compounds 1–8

The photocatalytic reaction of reductive water decomposition promoted by **1**–**8** coordination polymers goes through both Dexter and Forster energy transfer mechanisms. Since Forster and Dexter mechanism usually proposed for supramolecular assemblies having weak coupling interaction between chromophores, these mechanisms more suitable describe energy transfer in host-guest coordination polymers. Specifically, the energy transfer by Forster mechanism takes place via dipole—dipole interactions, *i.e*. long-range dipole–dipole energy transfer, in which the transition dipoles of donor and acceptor would couple with an inverse sixth-power dependence on the separation [1]. Furthermore, the energy transfer by Dexter mechanism introduces an electron exchange interaction where the rate of energy transfer depends on the distance between the donor and acceptor units [2].

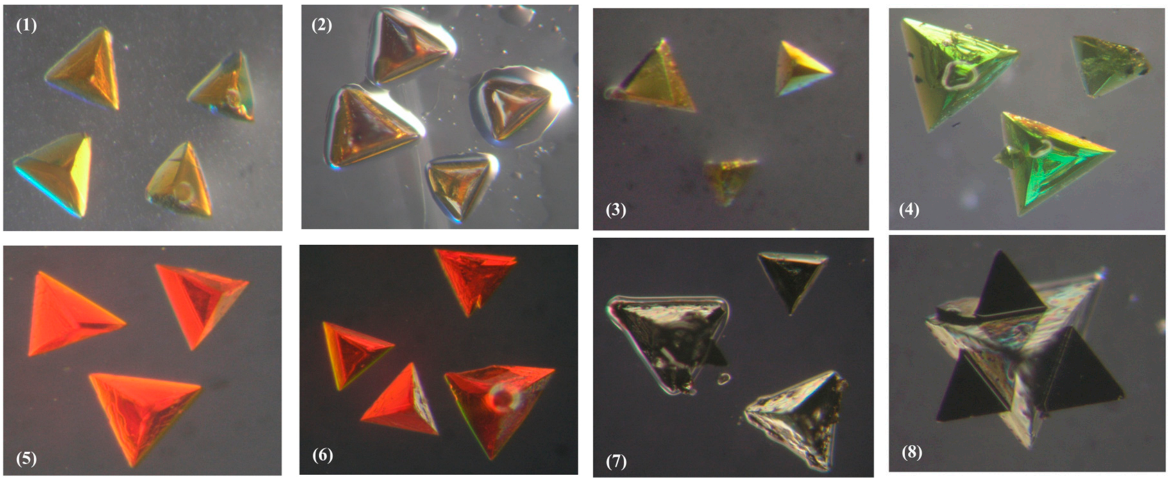

Figure S1. Digital photographs showing the crystal habits of compounds **1**–**8** grown in gel media.

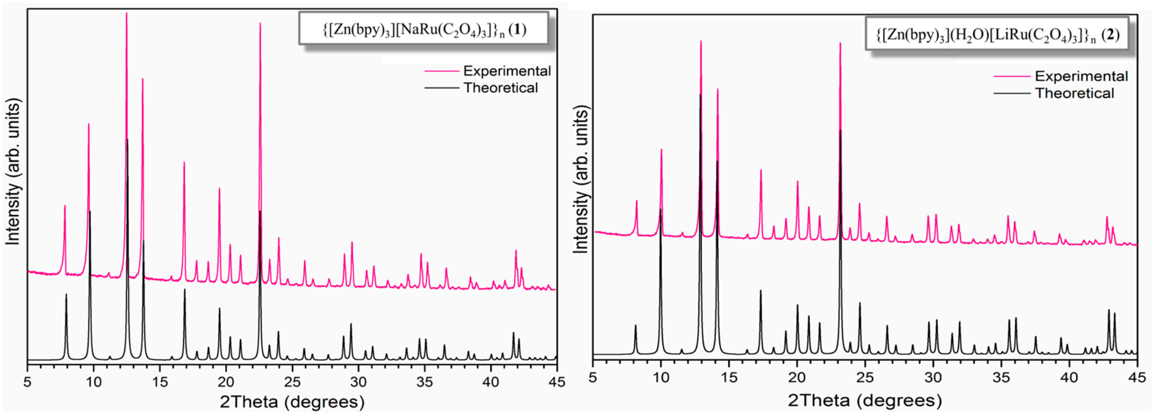

Figure S2. Experimental and theoretical powder X-Ray diffraction patterns for $\{[Zn^{II}(bpy)_3][NaRu(C_2O_4)_3]\}_n$ (**1**) and $\{[Zn^{II}(bpy)_3](H_2O)[LiRu(C_2O_4)_3]\}_n$ (**2**), indication the phase purity.

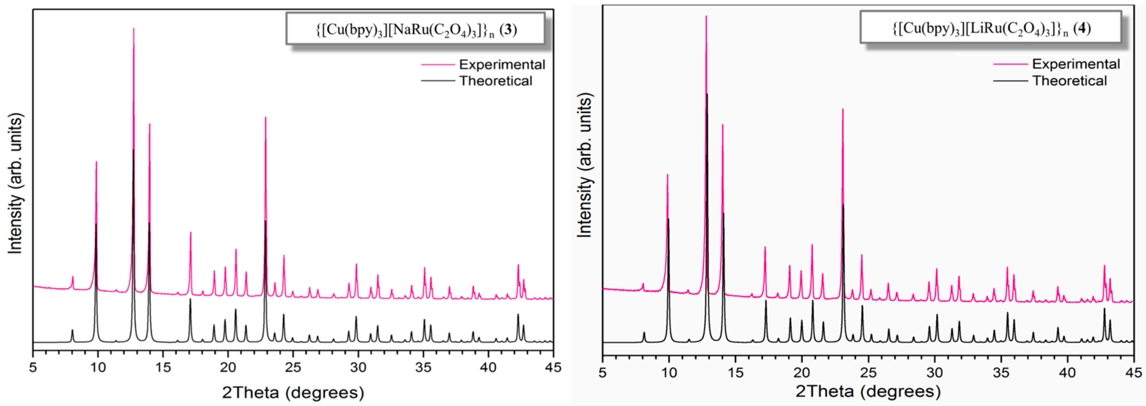

Figure S3. Experimental and theoretical powder X-Ray diffraction patterns for $\{[Cu^{II}(bpy)_3][NaRu(C_2O_4)_3]\}_n$ (**3**) and $\{[Cu^{II}(bpy)_3][LiRu(C_2O_4)_3]\}_n$ (**4**), indication the phase purity.

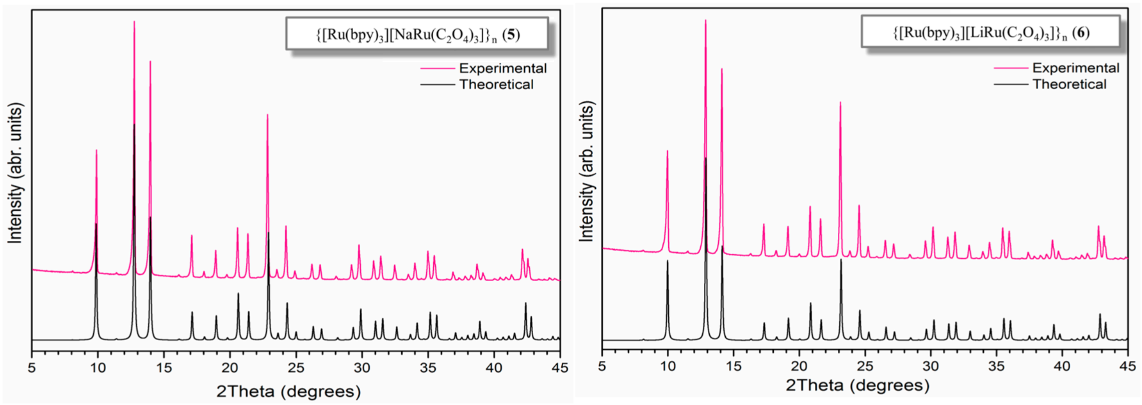

Figure S4. Experimental and theoretical powder X-Ray diffraction patterns for $\{[Ru^{II}(bpy)_3][NaRu(C_2O_4)_3]\}_n$ (**5**) and $\{[Ru^{II}(bpy)_3][LiRu(C_2O_4)_3]\}_n$ (**6**), indication the phase purity.

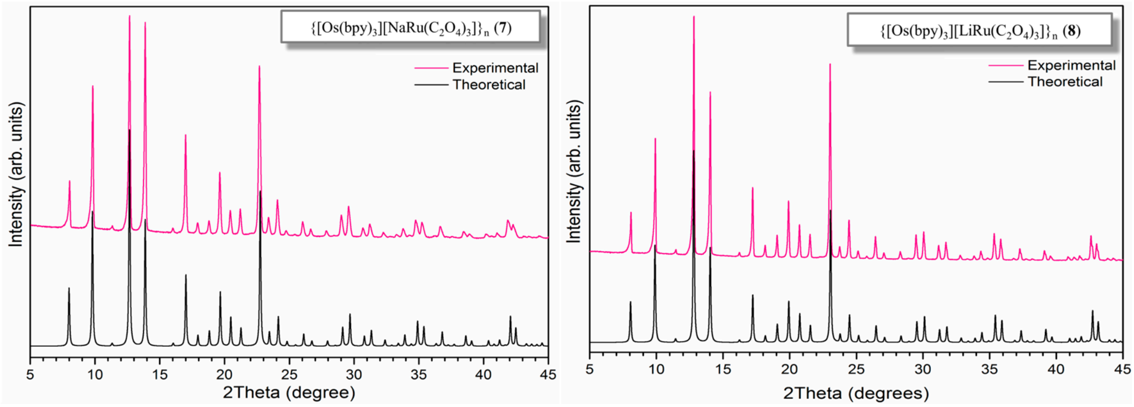

Figure S5. Experimental and theoretical powder X-Ray diffraction patterns for $\{[Os^{II}(bpy)_3][NaRu(C_2O_4)_3]\}_n$ (**7**) and $\{[Os^{II}(bpy)_3][LiRu(C_2O_4)_3]\}_n$ (**8**), indication the phase purity.

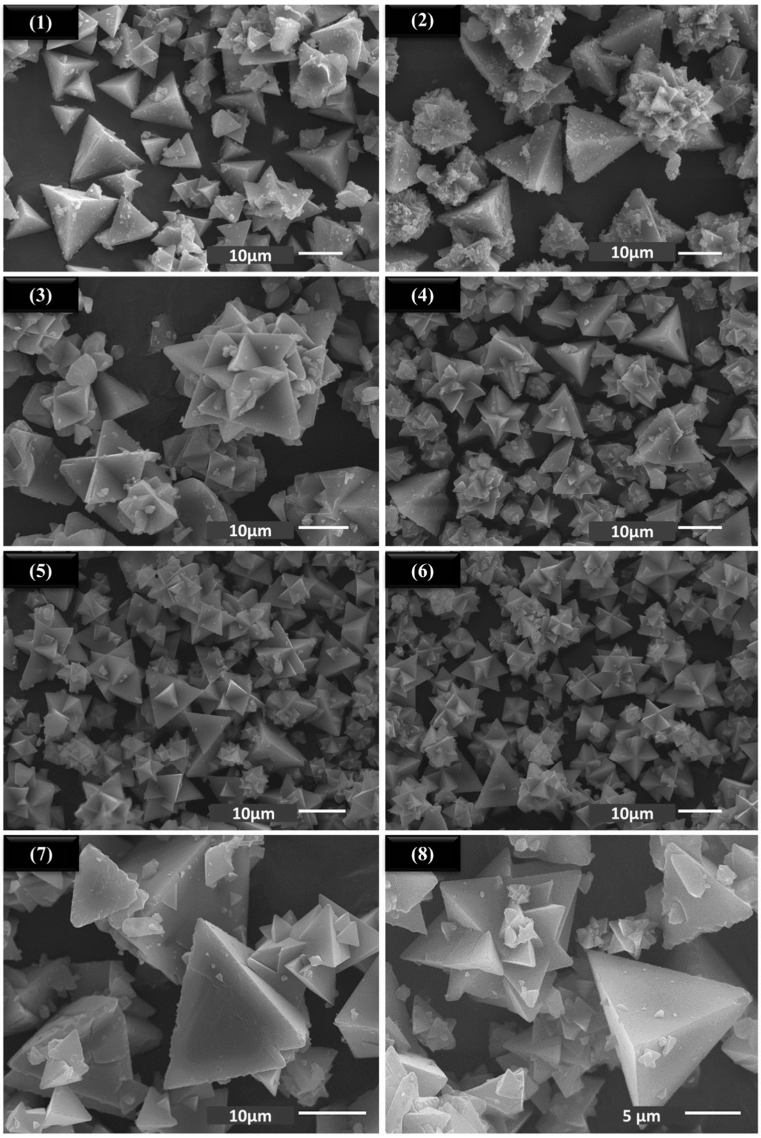

Figure S6. SEM images of compounds **1–8**.

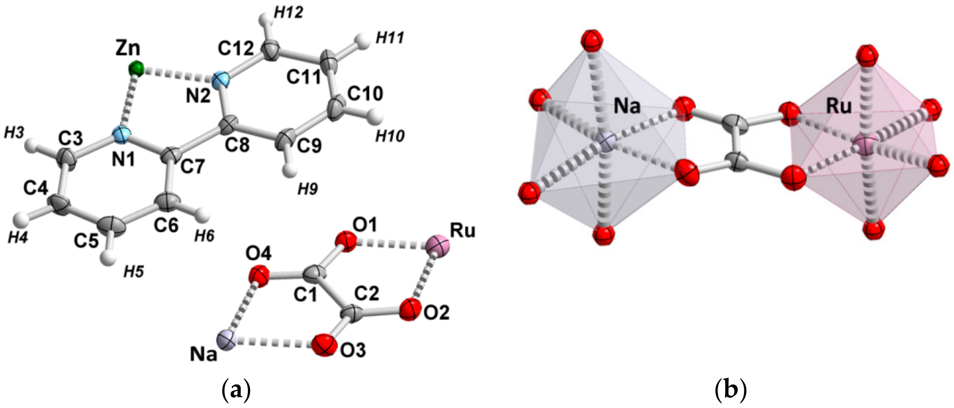

Figure S7. (**a**) View of the asymmetric unit of **1** with corresponding atom labeling scheme drawn with ellipsoids at the 50% probability level. (**b**) The bridging mode of oxalate ligand, Ru(III) and Na(I) coordination environment in compound **1**.

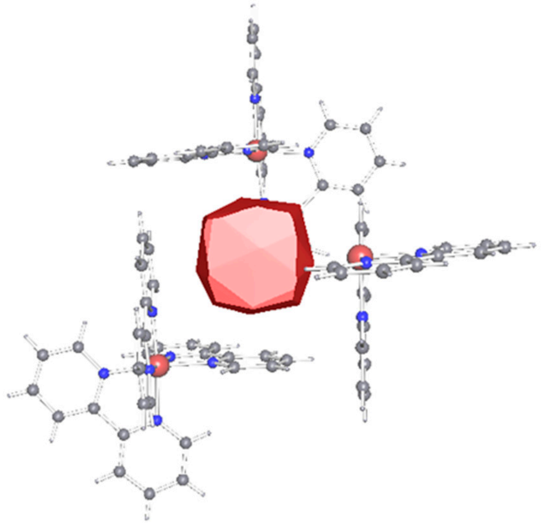

Figure S8. Voronoi-Dirichlet polyhedra representing volume of cubic bpy-cage ocupied by water molecule in **2**.

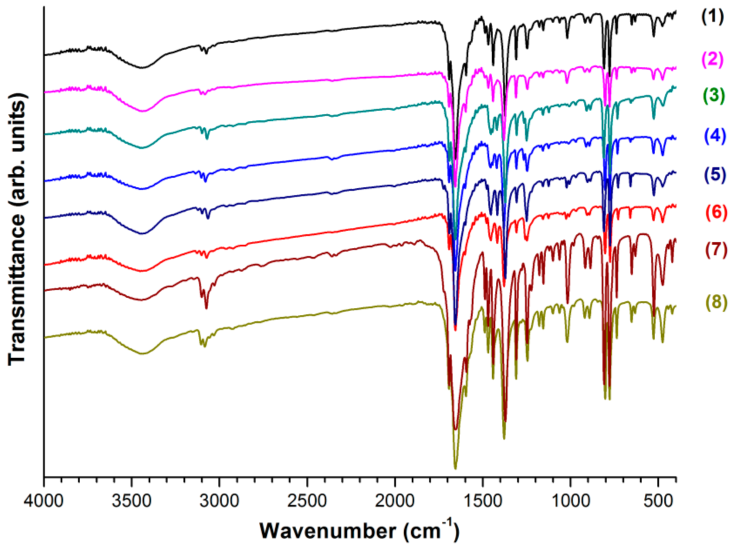

Figure S9. IR spectra of compounds **1**–**8**.

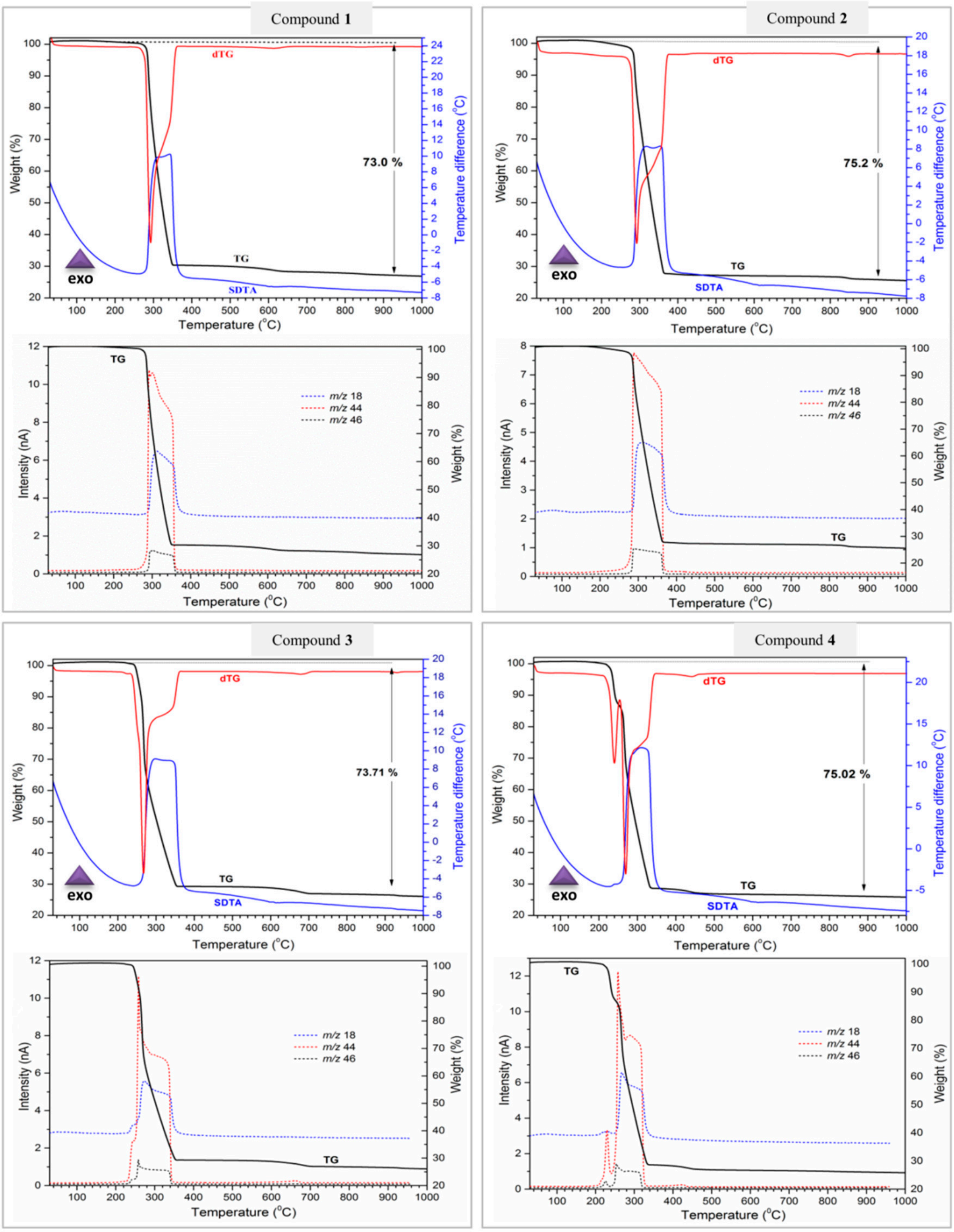

Figure S10. Thermal decomposition (TG, dTG, SDTA) and mass spectrometry of evacuated vapors curves of compounds **1**–**4** in air atmosphere.

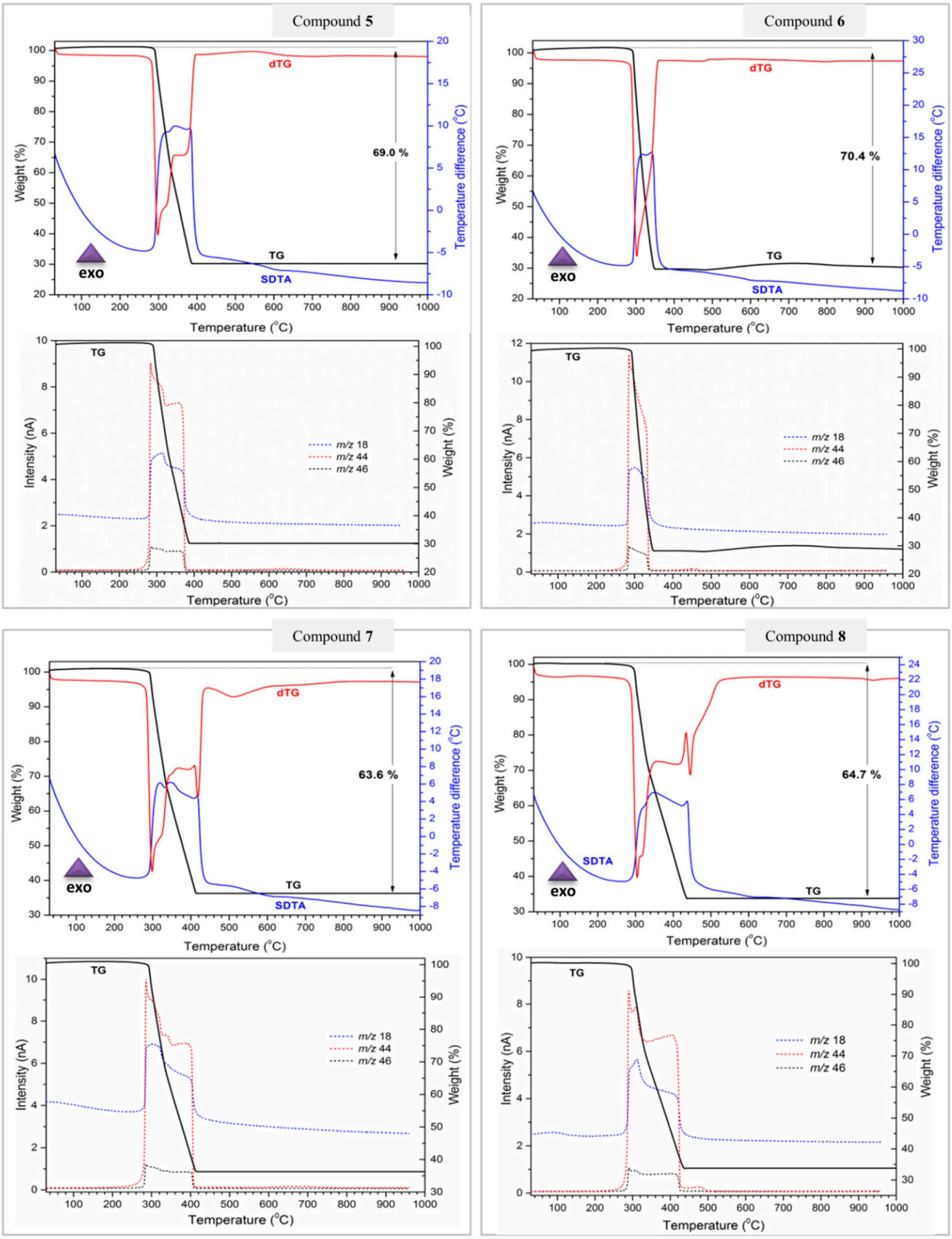

Figure S11. Thermal decomposition (TG, dTG, SDTA) and mass spectrometry of evacuated vapors curves of compounds **5–8** in air atmosphere.

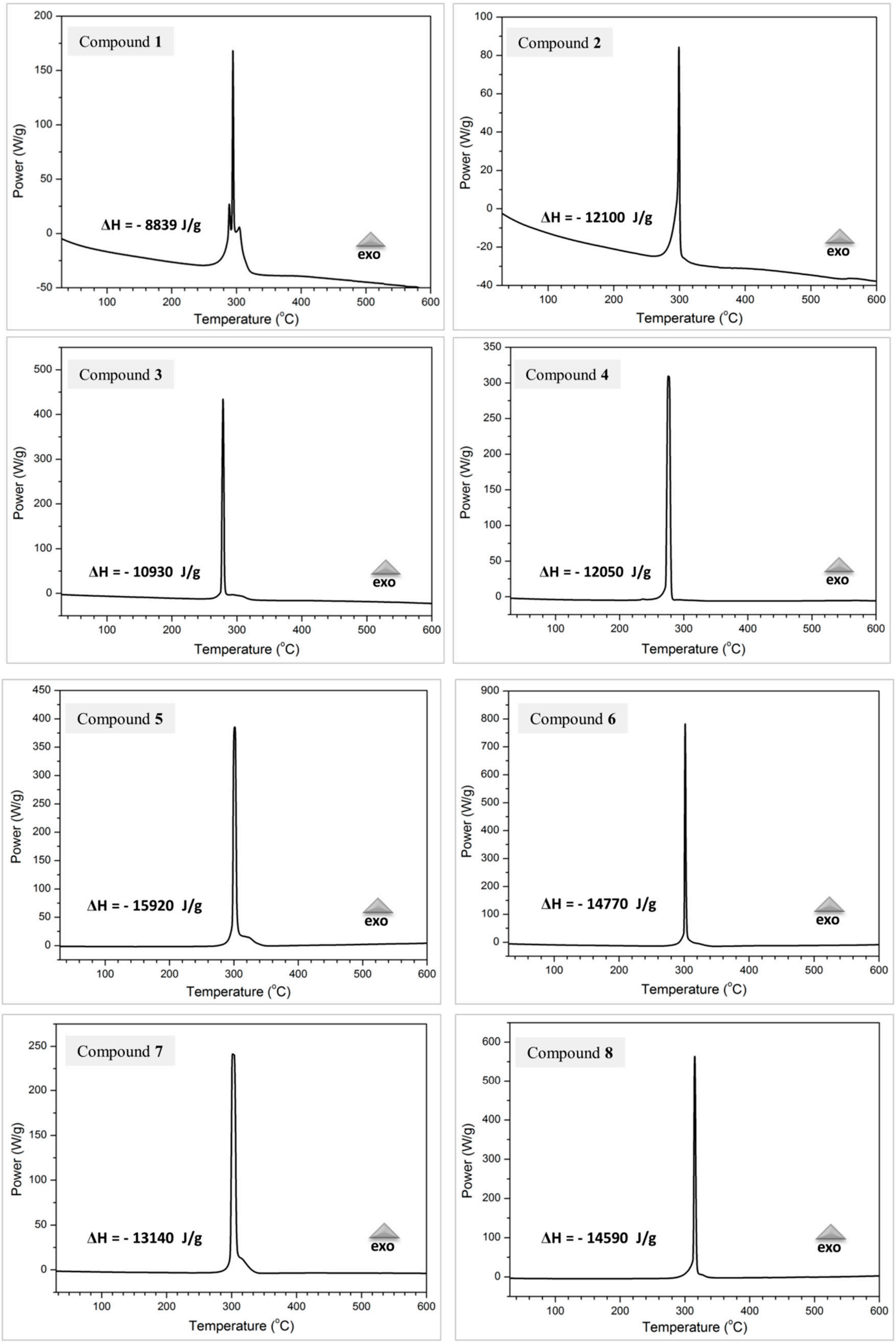

Figure S12. The DSC curves corresponding to thermal decomposition of compounds **1**–**8** in air atmosphere.

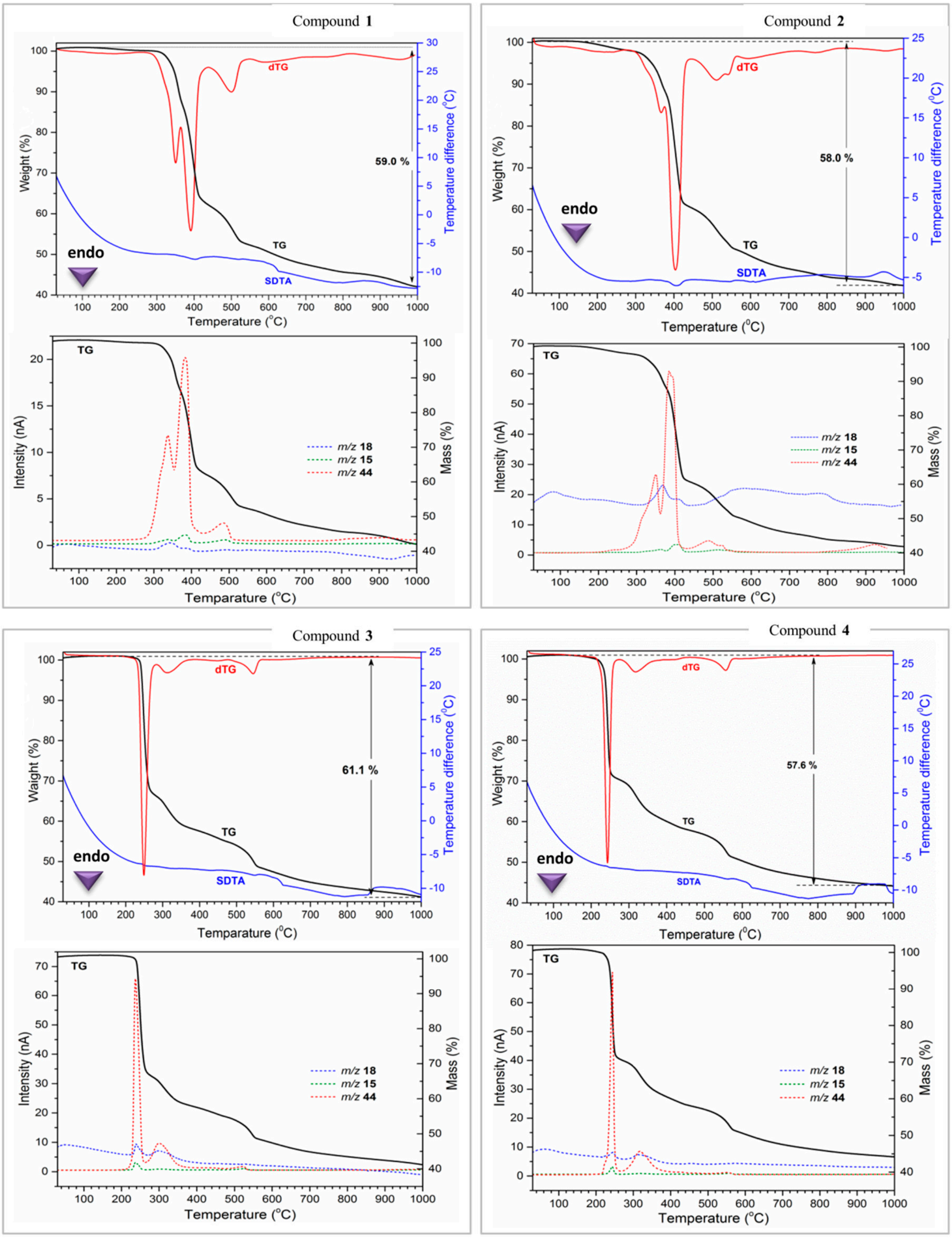

Figure S13. Thermal decomposition (TG, dTG, SDTA) and mass spectrometry of evacuated vapors curves of compounds **1**–**4** in nitrogen atmosphere.

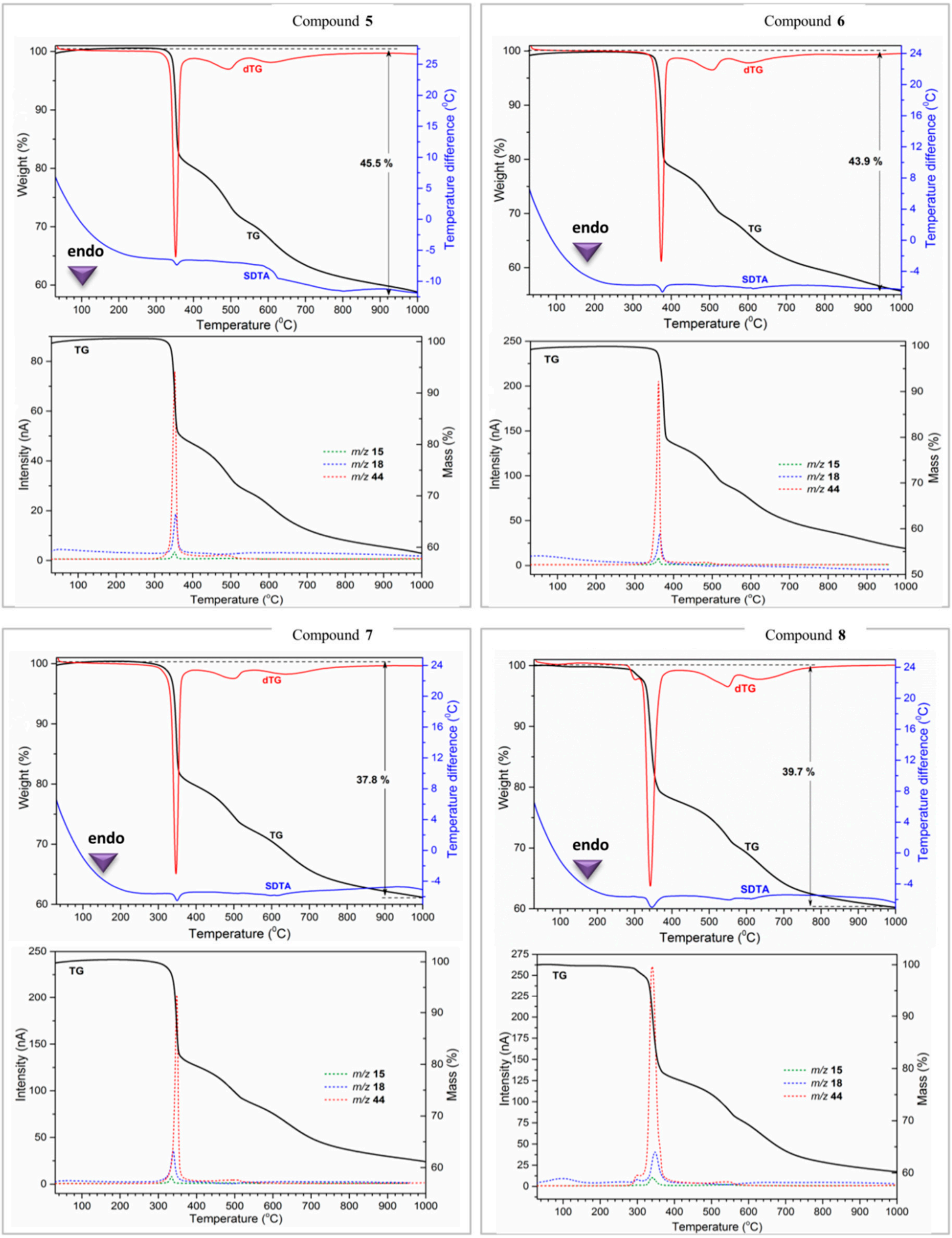

Figure S14. Thermal decomposition (TG, dTG, SDTA) and mass spectrometry of evacuated vapors curves of compounds **5**–**8** in nitrogen atmosphere.

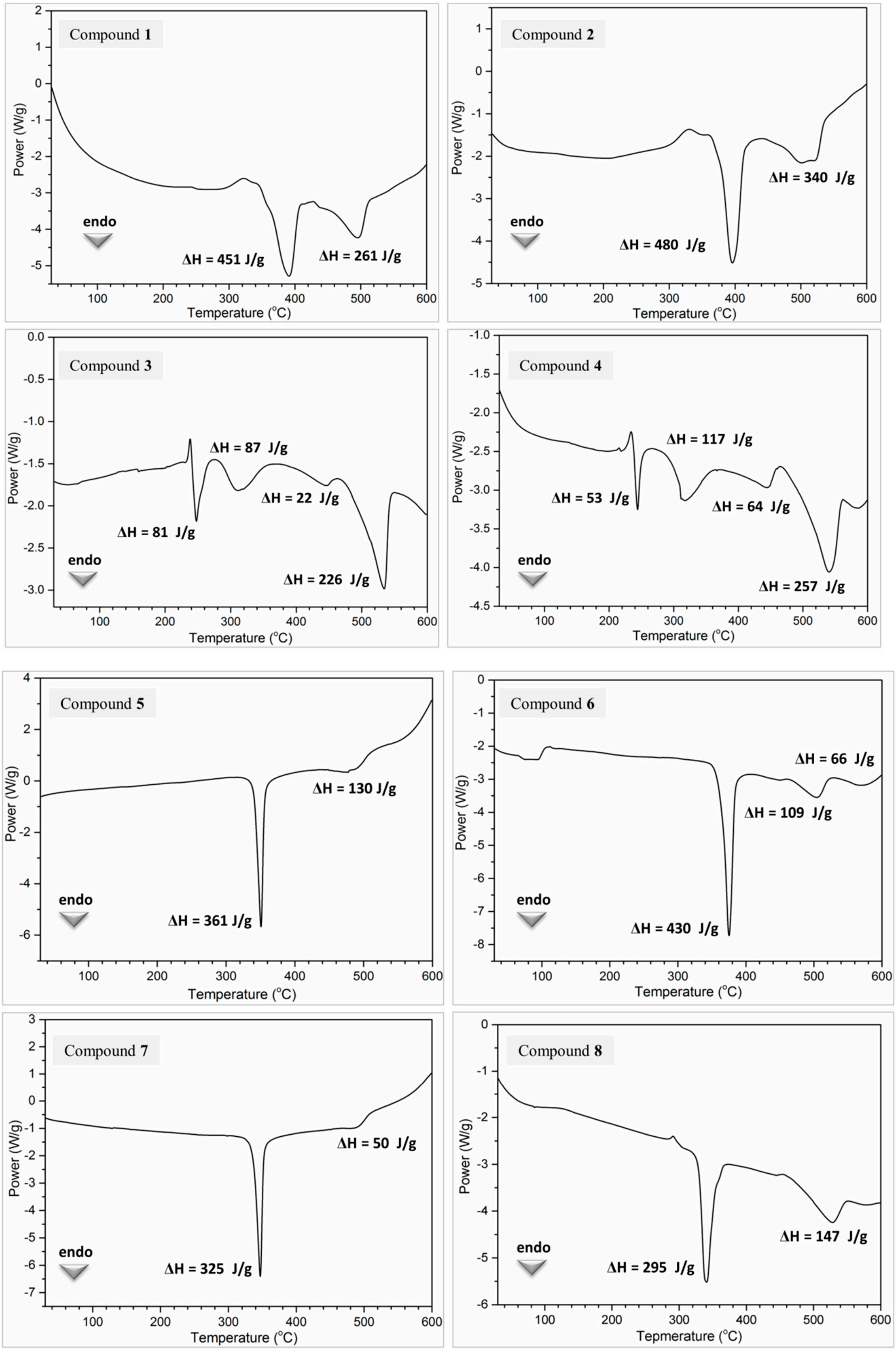

Figure S15. The DSC curves corresponding to thermal decomposition of compounds **1**–**8** in nitrogen atmosphere.

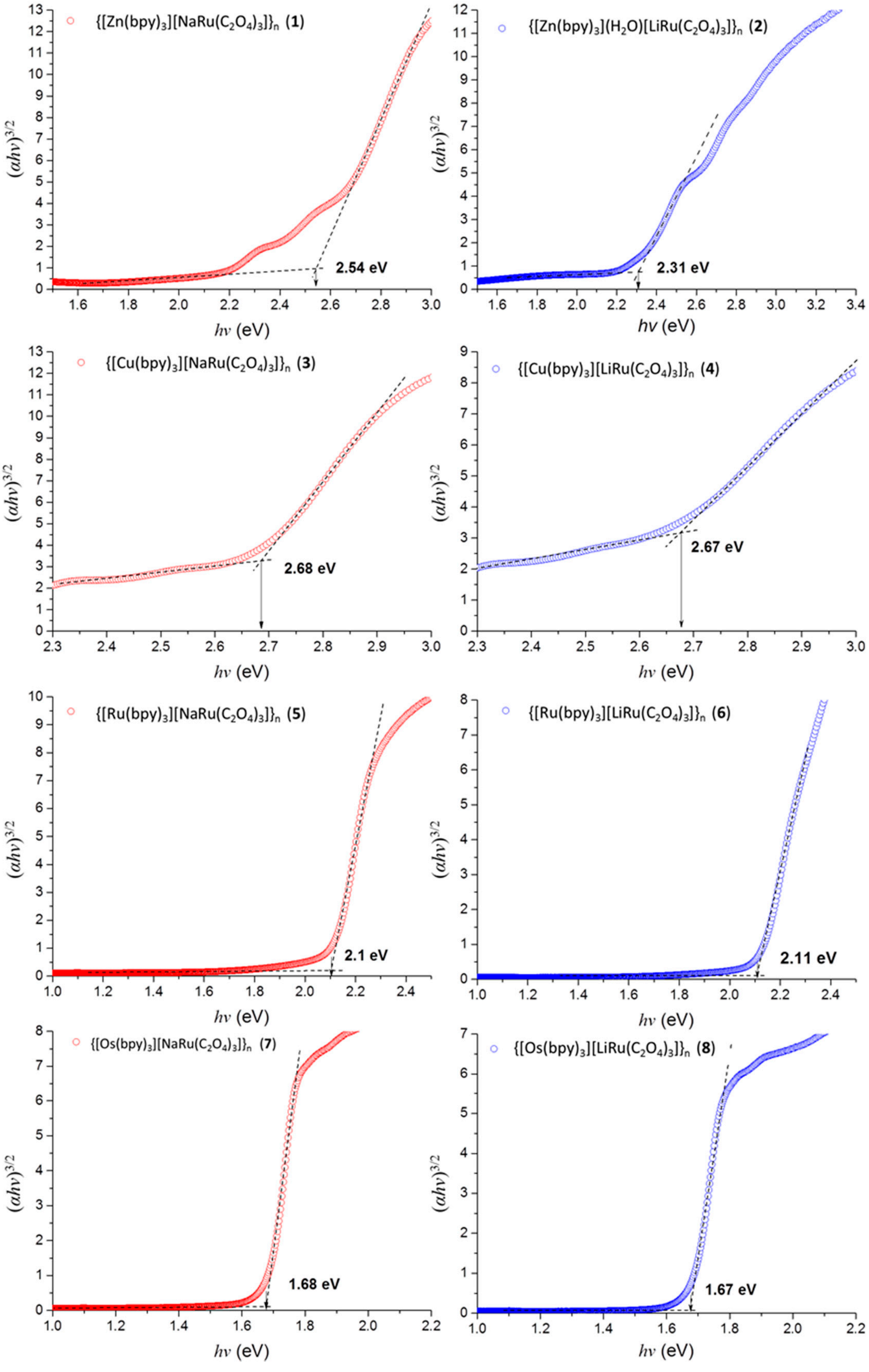

Figure S16. Tauc plots of Kubelka-Munk-transformed diffuse-reflectance spectra of compounds **1**–**8**.

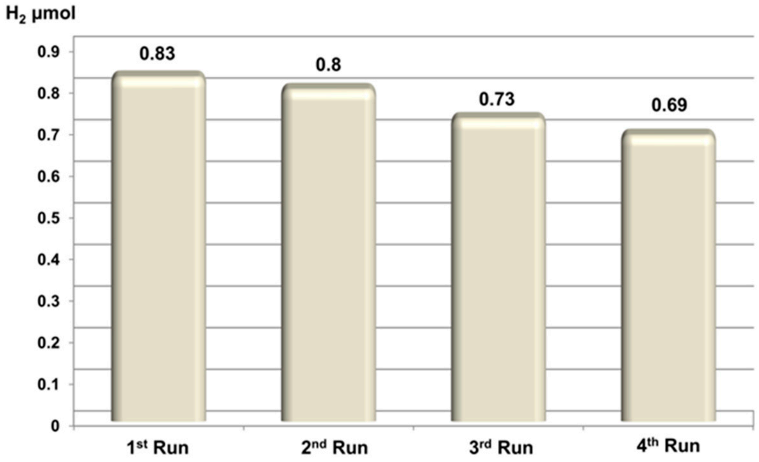

Figure S17. Catalytic activity of {[Ru^{II}(bpy)$_3$][LiRu(C$_2$O$_4$)$_3$]}$_n$ (**6**) in H_2 evolution during water photo-splitting reaction for four consecutive runs.

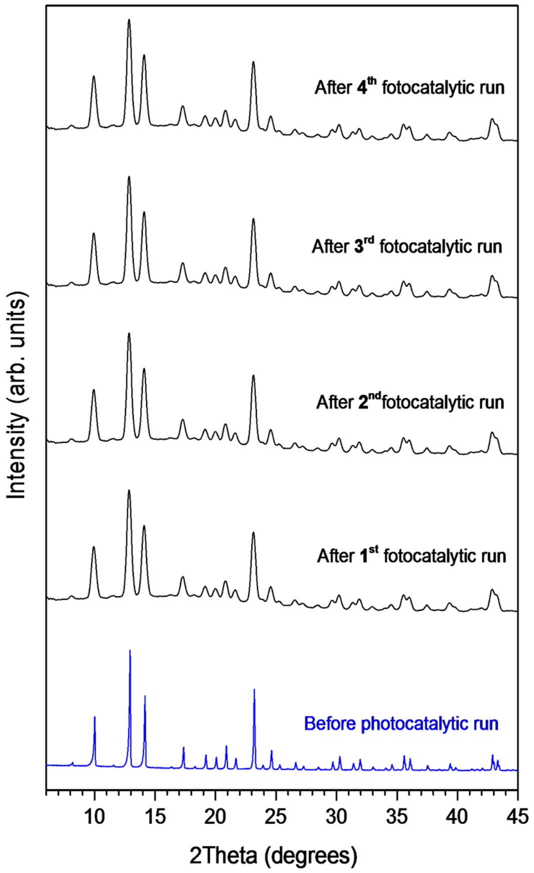

Figure S18. Comparison of powder X-Ray diffraction patterns of compound {[Ru^{II}(bpy)$_3$][LiRu(C$_2$O$_4$)$_3$]}$_n$ (**6**) before and after each repeated photocatalytic runs (four consecutive cycles).

Reference

1. Főrster, T. 10th Spiers Memorial Lecture: Transfer mechanisms of electronic excitation. *Discuss. Faraday Soc.* **1959**, *27*, 7–17.
2. Dexter, D.L. A Theory of Sensitized Luminescence in Solids. *J. Chem. Phys*. **1953**, *21*, 836–850.

© 2016 by the authors; licensee MDPI, Basel, Switzerland. This article is an open access article distributed under the terms and conditions of the Creative Commons by Attribution (CC-BY) license (http://creativecommons.org/licenses/by/4.0/).
